# Current scenario of solid waste management techniques and challenges in Covid-19 – A review

**DOI:** 10.1016/j.heliyon.2022.e09855

**Published:** 2022-07-02

**Authors:** J. Nimita Jebaranjitham, Jackson Durairaj Selvan Christyraj, Adhimoorthy Prasannan, Kamarajan Rajagopalan, Karthikeyan Subbiahanadar Chelladurai, Jemima Kamalapriya John Samuel Gnanaraja

**Affiliations:** a Department of Chemistry, Women’s Christian College (An Autonomous Institution Affiliated to University of Madras), College Road, Chennai 600 006, Tamil Nadu, India; b Regeneration and Stem Cell Biology Lab, Centre for Molecular and Nanomedical Sciences, International Research Centre, Sathyabama Institute of Science & Technology (Deemed to Be University), Chennai, Tamilnadu, India; c Department of Materials Science & Engineering, National Taiwan University of Science and Technology, Taipei, Taiwan, ROC; d Department of Biotechnology, Vel Tech Rangarajan Dr. Sagunthala R&D Institute of Science and Technology, Chennai, Tamilnadu, India

**Keywords:** Solid waste, Generation rate, Countries investment, Government policy acts, Covid-19

## Abstract

Annually, world generates 2.01 billion tonnes of solid wastes and it is expected to generate 2.2 billion tonnes of solid waste by 2025. Globally double the amount of waste generation was anticipated by 2050, hence an urgent action is required for this intricate problem in adopting better management techniques and recycling strategies. Unfortunately, poor management of wastes causes vulnerable effects to the society in terms of health. Waste management is the key infrastructure to be developed in society, but so far it is not recognized as much in many developing countries. Significant innovations and improvements are made in the last few decades globally, but still 2 to 3 billion people around the world lack access to waste collection services. The aim of this present study is to give an overview of different types of waste techniques that are effectively followed by different countries and the action plans need to follow. This review focuses on the global current scenario of waste generation, and its management methods with relevant literatures providing the upgrades in the phases of waste management services like collection and transport, various techniques adopted for waste management, policies and legislation, countries investment in waste management process and the impact of solid waste management during Covid-19. Collectively we conclude that Asian countries need to allot more fund for handling solid waste. Also with the available waste management technique, it is not possible to achieve zero waste. Therefore, more new techniques are needed to be adapted.

## 1 Introduction

**Figure 1:**
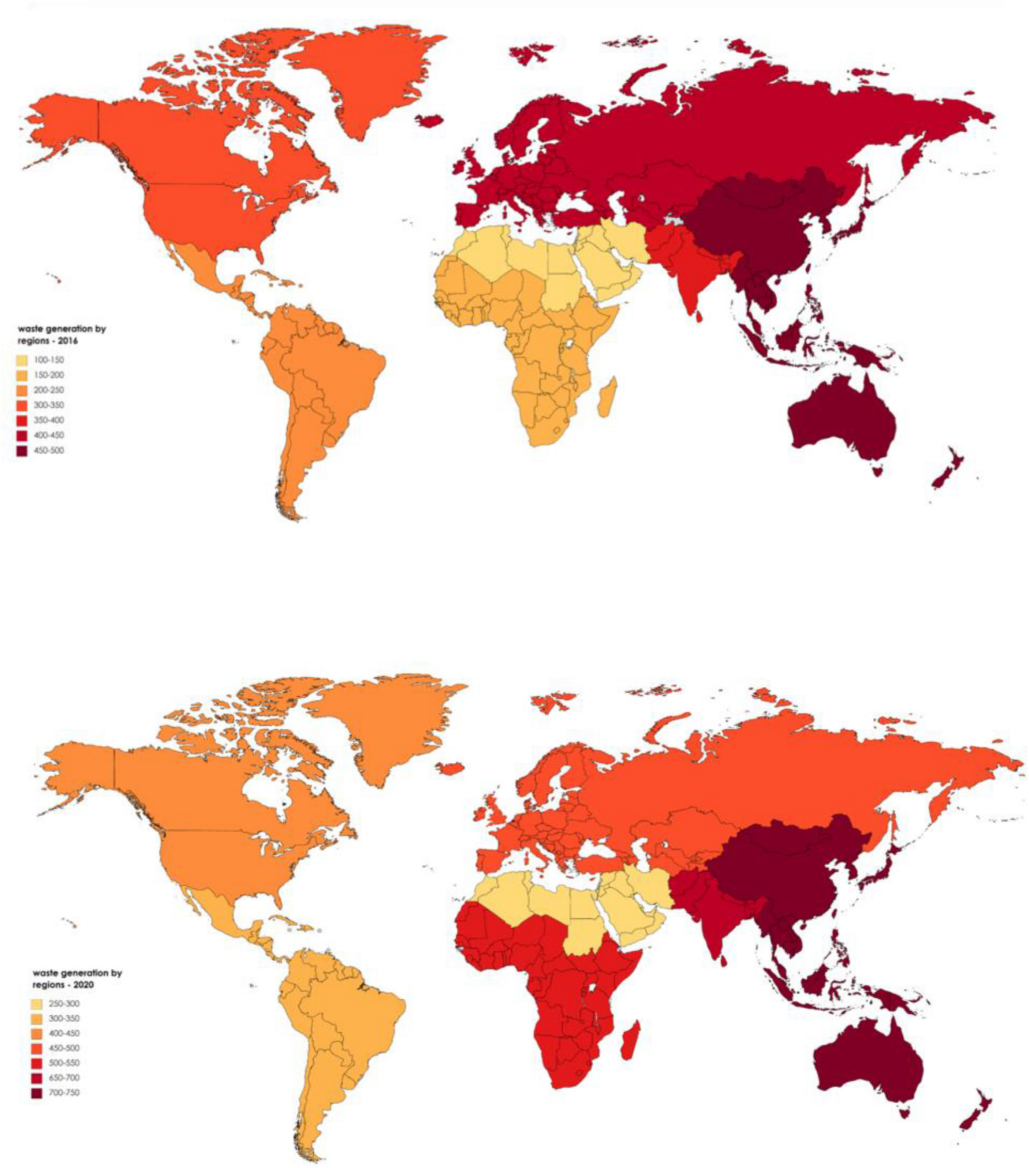
a, b worldwide scenario of total waste generation (in millions of tons) in year 2016 and projected waste generation for the year 2050. A. The maximum quantity of waste (450–500 million tons) generated is reported from East Asia and Pacific regions and the least quantities of waste is reported from the Middle East and North African regions. B. Based on the recent projected report on waste generation for the year 2050, the maximum quantity of waste (700–750 million tons) is reported to be high in East Asia and Pacific regions. The Middle East, North Africa, East Asia and pacific had no significant increasing pattern of waste generation when compares it with the 2016 data. But significant quantity of waste generation will be reported in Sub Saharan Africa and South Asia rather than Latin America and Caribbean, North America, Europe and Central Asia in the future year of 2050

Managing solid waste is a challenging task all over the world and the major reason behind it is lack of social awareness, responsibilities together with a lack of novel solutions. Globally, it is noted that the waste generation rate increase with increasing income in other terms global urbanization paves the way for more waste generation (Kaza et al., 2018). In the past three decades, generation of the industrial and hazardous waste shifts more burden on developing countries. Increase in population, migration to nearby cities, increasing city sizes and development of new cities also increases the waste per capita of developing countries. Another reason behind the waste heap is due to lack of coverage in waste collection and as per the recent report around 2 billion people are unable to regularly access waste collection throughout the world (Besen and Fracalanza, 2016). Hence, improper management of waste can impose negative impact on public health, environment, productivity, tourism, economic status and damages associated with floods. Financial affordability is the biggest challenge for developing countries in handling solid waste and it needs much attention on cost-effective waste treatment techniques (Ferronato and Torretta, 2019). In another phase, the worldwide scenario of total waste has reached more than 2.01 billion of tonnes in 2016 (Kaza et al., 2018). The maximum quantity of 450–500 Mt waste generation was reported from East Asia and Pacific regions and the least quantities of waste was reported from the Middle East and North African regions. Based on the projected report on waste generation, 2050 may explore the maximum quantity of waste (700–750 million tons) will be high in East Asia and Pacific regions (Kaza et al., 2018). The Middle East, North Africa, East Asia and Pacific had no significant increasing pattern of waste generation when compared with 2016 records. But significant quantity of waste generation will be reported in Sub Saharan Africa and South Asia rather than Latin America and the Caribbean, North America, Europe and Central Asia in the future year 2050 (Kaza et al., 2018). Every year, India has generated 65 million tonnes of waste. According to the report of the Ministry of Housing and Urban Affairs (MoHUA) 2020, the total waste generation level by Indian states (84,475 wards) was reported as 14, 7613 Mt/d (Singh, 2019). The current and future views of worldwide solid waste generation was shown in Figure 1a, b. To implement the new ideas to manage the solid wastes in India, a report has been submitted by the parliamentary ‘Standing committee on Urban Development’ on Solid Waste Management including Hazardous Waste, Medical Waste and E-Waste in 2019. At the same time, the “action taken report” was also submitted in the parliament in March 2021, among 21 actions, only 14 actions have been accepted by the government and 2 actions are still awaited by the committee for approval (Pavithra, 2021). Recent literatures states that, the solid waste management is still in unsustainable condition notably as developing and underdeveloped countries are highly affected (Ntagisanimana et al., 2021). Initially, the influence of Covid-19 lockdowns towards the generation of solid waste (Shrestha et al., 2020) was positive but on later stages biomedical waste generation have been increased uncontrollably in the form of Personal Protective Equipment (PPE) equipment’s (Das et al., 2020). In general, two solutions are available for the reduction of wastages viz., no production of wastages or converting the wastages into another material. In current scenario, reaching zero waste is impossible because of the industrialization and urbanization but using an effective waste management, it is possible to control the waste. The effective waste management system will help to attain the future visions of complete recycling in such a way that waste materials could be used again as feed for another industry that will help to reduce the levels of waste. Generally, Solid Waste Management concerns the application of the principle of Integrated Solid Waste Management (ISWM) to municipal waste (Ministry of Urban Development-India, 2013). Understanding the key composition of waste is very important for proper handling of the waste. The composition of solid waste includes food waste, paper, plastic, metals. glass, textiles, rubber, leather, construction and demolishing wastes. Also, in general plastic, paper and other packaging waste are highly generated in higher income countries such as Japan, Germany, UK, France, Italy, USA, Canada, Brazil, Turkey, China (Tisserant et al., 2017). According to the income level notable differences have been noted in the municipal solid waste management (organic waste—highest rate of waste mass by 57.69%). When compared to higher income countries, the waste from developing countries are produced more than half of total solid waste, whereas, the lower income countries such as North Africa, Sub-Saharan Africa (Kaza et al., 2018) had more organic content, high moisture, high density and lower calorific value which makes it easy to handle. Characteristics of Municipal Solid Waste Combustion (MSWC) has varied in summer and winter sessions that also reveals the relationship between the income level and organic waste viz., the income level is inversely proportional to the organic waste because the high-income countries approximately 44,000 tons of package of waste will be thrown with other solid waste. It indicates the demand for amelioration of recycling process in high level income countries (Ozcan et al., 2016). However, this concept of the review gives an overview about the recommendations for controlling the waste generation, amelioration of energy recovering process and proper awareness to the people that will formulate the society. We have referred to more than 150 articles and collectively reported the actual and future scenarios of waste management in all contexts, we hypothesize that there are no well-established modern techniques available to achieve the goal of zero waste. Waste can be managed to some extent with the available techniques, but there is a financial barrier for economically lower countries. Waste management challenges are everywhere and that is initiated from the housing societies itself, for an example, no segregation at source, inadequate segregation techniques, slow adoption of in-house composting, lack of monitoring in housing societies etc., World governments should educate and the action plans of waste management to the common people. Specially, the East Asian and Pacific countries should execute the action plans and fix the issues immediately for avoiding the generation rate. Positively, 52.32% waste materials are biodegradable but 33% of waste are disposed by open dump, this should be fixed by executing the 3Rs (Reduce, Reuse and Recycle) technology. Notably, the recycling treatment should be improved in some of the countries which follow only less than 10% of recycling systems such as, South Asia, East Asia and Pacific Middle East and North Africa. In general, some of the waste materials decomposing time is more than years so government should strictly fix the time of decomposing of waste materials and should prohibit those materials.

## 2 Waste management—overview

**Figure 2:**
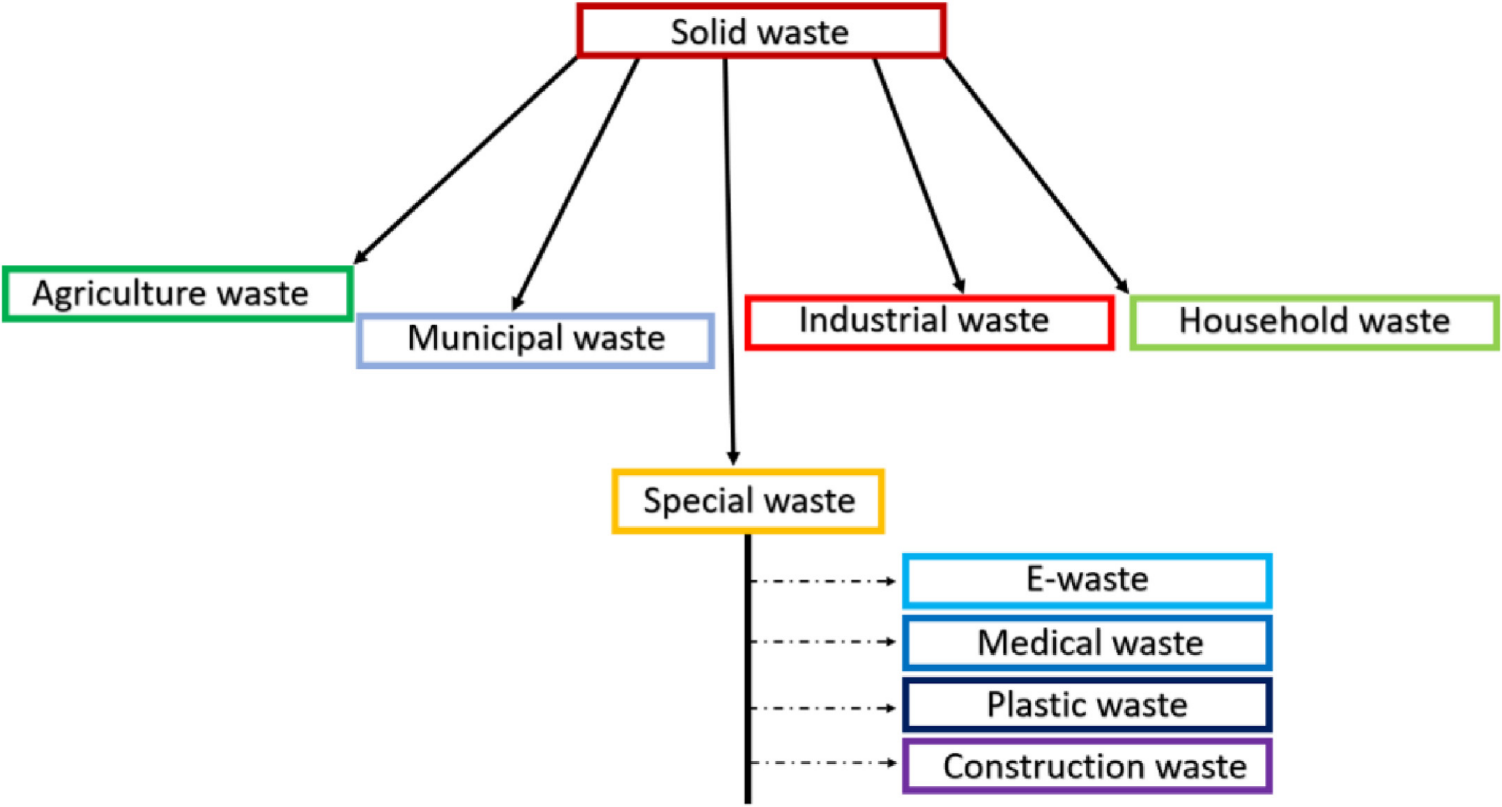
Categorization of Solid Waste. Viz., agricultural waste, municipal waste, industrial waste, house hold waste and special waste (e-waste, medical waste, plastic waste and construction waste.

Generally, solid waste categorized into agricultural waste, municipal waste, industrial waste, house hold waste and special waste (e-waste, medical waste, plastic waste and construction waste) have shown in Figure 2. Plastic is widely used material around the world for different purposes and its usage is merged with people’s everyday life. The rapid urbanization and the modernization led to larger usage of plastic material and mostly plastics were used once (single-use plastics) and are immediately discarded. The greatest environmental health effect was caused by plastic waste, viz., 6.3 Mt of plastic waste have been generated in 2018 itself but only 9% was recycled and 12% was incinerated (Alabi et al., 2019). The population and future waste generation at Kolkata (Indian Metropolitan City) in 2021 have been projected as 16105614.85 and waste generation per day is about 6,442 Mt/day likely in 2031, the population will be 17870628.33 and waste generation per day is about 8,042 Mt/day and in 2035 the population will be 17870628.33 and waste generation per day is about 8,805 Mt/day (Das and Bhattacharyya, 2014). Globally plastic reached 330 Mt of its production in the year of 2016 (Eco-profiles, 2021). In the next 20 years, it was expected to double. Less than a quarter of the waste is being collected and treated. The mismanaged plastic waste (MPW) accumulated around 60 to 99 Mt globally in 2015 and this quantity can be tripled to up to 155–265 Mt y−1 by 2060 (Lebreton and Andrady, 2019). Most of the plastic wastes were washed into the ocean, burned by incinerators and the residues or source is dumped in landfills. These huge volumes of plastic waste leads to very serious consequences, such as environmental pollution, food chain contamination, energy waste, biodiversity breakdowns, economic loss, impacts on wildlife with bioaccumulation of synthetic contaminants and plastic elements (Nnorom and Odeyingbo, 2020). There are four strategies to manage the plastic wastes, that are (A) landfilling, (B) incineration, (C) reduce, reuse, recycle, (D) Create awareness among the people. Incineration decreases the need for landfill of plastic waste but not every country has the infrastructure. In global level, recycling rates of plastic waste is only about 14–18%. Remaining plastic waste is either processed for incineration (24%) or disposed as a landfill (58–62%) (OECD, 2018). Incineration is an inevitable option for the treatment and reduction of 95–96% of plastic waste. In Sweden 49% of waste are burnt for energy needs and in which 8% are from the incineration of plastic waste (Future, 2022). Incineration of plastic waste results with the emission of acid gases, particulates, nitrogen oxide, carcinogen dioxin, Polyvinyl chloride (PVC) and heavy metals are produced and it severely affect humans as well as animals (Manisalidis et al., 2020). Notably, in human it causes birth defects, cancer, neurological problems, reproductive dysfunction, etc., Polyvinyl chloride (PVC) emission is the main reason for toxicity and usage of alternate form of PVC is encouraged that will reduce the toxin level and effect of plastics during incineration and landfill. Examples of PVC alternate that are used in the field of medicine include ambulatory products (steel), non-sterilized (polyolefin, polyethylene, polypropylene), blood products (silicon, stainless steel, Non-DHEP PVC), epidural catheters (polyamide, polyethylene, polyurethane), laparoscopy (polyurethane), intravenous (makrolon, glass, polystedecompr), gloves surgical (nitriles, polychloroprene, polyurethane), Sterilized bags (polyurethane, polyolefin, polyester). In regular lifestyle the plastic usage are minimized by shifting to non-plastic material like reusable shopping bags, compostable garbage bags, steel bottles, bamboo toothbrush, eco-friendly tumblers, cloth diapers, cloth pads, wooden spoons, bamboo plates, reusable straws, aluminium foil for food packaging. Electronic and electrical waste (e-waste) is a kind of waste that are generated from electrical equipment failing its life time or discarded for the reason of new up gradation in new devices. Components of electrical and electronic equipment includes circuit boards, batteries, cathode-ray tubes, plastic casings, activated glass, and lead capacitors which are also classified in the category of e-waste. According to the recent global survey, generation of e-waste has been increasing at the rate of 2-million-ton metrics per yr (2014—approximately 41 million tonnes e-waste have been generated (Kumar et al., 2017), which equates generation of 49.8-million-ton metrics in 2018. The e-waste is highly hazardous to human health and it exhibit as inherent mutagen in all living population (Alabi et al., 2012). The Global E-Waste Monitor report in 2020 have revealed that 54 Mt of electronics have been discarded and only 17% of it was recycled. India generated 3.2 million tonnes of e-waste last year, ranking third after China (10.1 million tonnes) and the United States (6.9 million tonnes). Notably, China have generated 10.1 Mt of e-waste in 2019. Secondly, US produced 6.9 million waste and India generates 5.6 million in the same year of 2019. Importantly, ASSOCHAM-EY joint report estimation of Electronic Waste Management in India have predicted India will generate 5 million tonnes of e-waste in 2021 (Bhagat-Ganguly, 2021). The only key to resolve the issue is to implement the advanced recycling process on e-waste management systems as well as the ameliorating the energy recovering process like aluminium products from e-waste to increase the economic status of the country (Namo e-waste) (Dubey, 2019). E-waste recycling process consists of the succeeding steps like collection from source site, pre-processing, recovery of reusable materials and final disposal of non-recyclable residues. Recycling of the e-waste is the best management systems to reduce the hazards. E-waste recycling can be a market-based activity to be adapted by all countries. But in overall 70% of toxic substances from waste are only came from electric waste materials. While recovering workers are aiming for gold, copper but they have a condition at a risk of exposure to over 1,000 harmful substances like arsenic, mercury, lead, cadmium, selenium, flame retardants and hexavalent chromium. Alternative of those chemicals could be an effective solution for e-waste like ameliorating wood-based circuit boards, Nano cellulose materials for the production of electric materials (Homegrown, 2018). Construction and demolition activities and the disasters trailed by natural calamities create massive quantities of building wastes. Construction and Demolition Wastes (CDW) include non-biodegradable and inert materials such as metals, concrete, wood, plastic, ceramics, asbestos, plaster, paints, solvent and aqueous solutions. About 35% of the construction and demolition waste that are raised globally are directly filled up in landfills without any treatment (Menegaki and Damigos, 2018). Until 2012, annually more than 3.0 billion tonnes of CDW were generated by 40 countries within six continents (Oceania, North America, South America, Asia, Europe and Africa) worldwide and this trend has been increasing constantly (Akhtar and Sarmah, 2018). Recover, recycle and reuse are the three basic rules to control or manage the waste generated through construction and demolition however, CDW are deposited directly to landfills. The worldwide policies are required to be recognized in order to reduce greenhouse gas emissions, climate change and resources depletion with a focus on recycling and by encouraging circular economy to ensure the sustainable use of construction materials. The waste quantities of construction waste are quite bigger in metropolitan areas and construction of new buildings (Barbuta et al., 2015). In future, major cities will be turn to metropolitan cities so the concern on the reduction and management of construction solid waste is very imperative. In United Kingdom, government are providing financial assistance for the recycling CDW waste in tie up with construction companies. Also, to prevent illegal dumping they are charging landfill tax which are also gradually increased in every annual year that will force them to shift to recycling of CDW (Osmani, 2011). The waste generated by health care facilities like hospitals, laboratories, medical research facilities, etc. called as medical waste (Safeopedia, 2018). Medical waste generation is rapidly rising worldwide which could be penitential risk for both humans and environment. As per World Health Organization around 85% of healthcare wastes are non-hazardous, 10% are infectious (wastes from infected patients, diagnostic samples, etc.) and the remaining 5% is hazardous healthcare waste (chemical, radioactive) (WHO, 2021). There are methods for treatment and disposal option for medical waste such as incineration, autoclaving, disinfectant, chemical treatment, and more on. However, the World Health Organization recommended incineration technology as a temporary method for the treatment of hospital waste. It is very important to know that in the process of waste management in lower income settings, the waste collection and transport consumes around 60–80% of expenditures of waste budget community (Singh et al., 2014). But in spite of its expenditure spend, it is worth when compared it with public health and other losses that happen due to non-management of waste. In developing countries, the waste collection or coverage is commonly less than 50% in most of the cities such as Dhaka (167 kg per capita), Nairobi (219 kg per capita), Quezon City (257 kg per capita), etc., (Habitat n.d. 2010; World bank group, 2012; Wilson et al., 2013). The waste generated by the person is 0.401 kg per day and only <50% of waste has been collected from the local landfill (Villalba et al., 2020). One of the Indian states, Chennai has generated 6683 tones (December, January, February) in the winter season and 5754 (March, April and May) tones in the summer season (Mozhiarasi et al., 2020). Regular and reliable waste collection service to every residence is very important for treatment and safe disposal of waste. Interestingly, recent reports reveals that 100% door to door waste collection has been achieved in most Indian cities for an example, Chennai and Mumbai have achieved 90% of door to door waste collection. Across 11 different states in India, the total average of wards with 100% door to door collection is 81,135 (96.05%) out of 84,7475 (Singh, 2019). Globally, the wastages are collected by two major processes such as primary collection (collection points—trucks will collect the wastages from people) and secondary collection (transportation of collected wastages to the treatment centres). As a latest upgrade instead of manual loading of the container in vehicles, a container is designed in such a way that can be automatically loaded into the collecting vehicles. In smart containers, sensors are used which helps to monitor the fill in levels of the container that aids in effective communication and unloading of waste. Choosing the appropriate vehicle is also an important factor for effective collection of waste. A small vehicle like motorized three-wheelers are able to cover the narrow street which makes it advantageous in high traffic cities. Broad roads and infrastructure are essential for handling large vehicles. Integrating technologies like Global Positioning System (GPS) enabled vehicles, Apps which supports real-time mapping of vehicles, additional services like collection of e-waste, plastic waste with a proper incentive can help to collect waste effectively. Monitoring and supervision are the key steps for efficient collection of waste from the source site. Several steps and context will be focused in the process of waste transferring systems like pre-planned vehicle routing, locating storage container in the appropriate place, determining the collection point, regular and reliable service, adapting techniques like GPS enabled vehicle, weighbridge, electronic on-board vehicle recorder and these are essential for efficient monitoring and supervision. The effectiveness of waste management has been often visible as an indicator of good governance (Yukalang et al., 2017). The public sector had a role in policy making and involvement in the translation of legislation in terms of protecting public health and the surrounding environment. The major challenges with the public sector are the divisions of power which makes it unclear in their roles and lacks coordination. Also, more often political interference, lower priority to the sub-urban areas, corruption, and lack of qualified staff, transparency and accountability makes the public sector extremely challenging. To develop staff capacities, collaboration with Non-Governmental Organization (NGO), local universities and training with the private sector in both governance and technology aspects can be more helpful to develop their skills (Delisle et al., 2005). A common network viz., Peer Experience and Reflective Learning (PEARL) was launched to strengthen the public sector in India from 2007 (PEARL, 2021). Different levels of private sector like community groups, joint-venture approaches with the public sector and other non-government organization are available as waste management systems. Allowing private sector’s participation in waste management will help to improve work efficiency, cost-effectiveness, regulatory flexibility and overall help the situation. As an example, through private sectors in Malaysia and Latin America the cost of waste management is reduced to a level of 20–50% (Kaza et al., 2018), Balanced partnership is an essential task to keep the healthy competition within the private sector as monopoly system may lead to increase in service cost at any time. The waste management law has been designed to eliminate or minimize the amount of waste materials that can affect the environment and economic status of the country. Legal services on solid waste management have been maintained by individual countries as well as worldwide countries for example., in United States of America (USA), they have various rules and legislations like US solid waste legislation—solid waste disposal act of 1965, Resource recovery act of 1970, Resource Conservation and Recovery Act of 1976 (RCRA) and the Hazardous and Solid Waste Amendments of 1984 (HSWA) to eliminate and minimize the production of waste and prevent the effects of solid waste on the health of the environment and economic status. Likewise, Convention on Civil Liability for Damage Caused during Carriage of Dangerous Goods by Road, Rail, and Inland Navigation Vessels, Geneva, 1989., FAO International Code of Conduct on the Distribution and Use of Pesticides, Rome, 1985 etc., The private sectors must obey the government laws and their agreements before planning for a partnership services, example, Sample Municipal Solid Waste (MSW) Public-Private Partnership (PPP) have two sections of solid waste viz., sample and contracts: Street cleaning and waste collection, Built-Operate-Transfer (BOT), Design-Build-Operate (DBO) and Concession agreements for municipal solid waste disposal, recycling, sanitary landfill and waste to energy. The PPP on waste management system is one of the best systems for the developing countries. The PPP is providing additional options to compare the management by contract. For an example, >70% residue of sanitary embankments has been generated than Municipal Corporation. It will ameliorate the public awareness on environmental education for people health as well as creating more job opportunities for the public by expanding the collector numbers from 50 to 600 (Marconsin and dos Santos Rosa, 2013). Importantly, these sample contracts have been designed by the World Bank and it’s designed for the construction of sanitary landfills and operation for the client countries. The major economic instruments of the solid waste management systems are disposal taxes, pollution taxes, eco-taxes, pollution charges, waste generation taxes, producer charges, waste tipping charges and product charges (Balasubramanian, 2020). To reduce waste management costs, it is important to think for 3R (Reduce, Reuse, Recycle) which ultimately helps to lower the equipment investment and disposal facilities cost (Petzet and Heilmeyer, 2012). Reduction is achieved by policy and as an example in India, Tamil Nadu banned single-use plastic bags from January 1, 2020 (Pollution, 2021). Reuse is practiced in many formats throughout the world for an example e-waste, paper and plastic waste are used to make as an artwork and more probability glass is reused to a large extend. Metals, glass, plastic and cardboard are most probably able to recycle multiple times. Recycling in developing countries is high through the participation of informal sectors. A report of Waste Statistics—Municipal Waste—Explanatory texts, 25 June 2012 stated that, Germany has used many advanced treatment techniques that ameliorated the probability of reuse and recycling up to 62% in 2010 (Srivastava, 2016). Waste treatment by means of dumpsite or landfill is not a sustainable solution because it has more side effects for humans and other animals (Swati et al., 2018). In general, some of waste treatment technologies have followed in world–wide viz., open dump, recycling, sanitary landfill, landfill unspecified, anaerobic digestion, composting, aerobic composting, controlled landfill, and incineration have shown in Figure 4. Land fill is the only option for many places for the destruction of waste, even in most of the cases after incineration it needs landfills, which affects ground water, surface water, environment and emit noxious smokes which have high potential to pollute the environment (Nai et al., 2021). The landfill causes a direct impact on water pollution (ground water by landfill leachates), air pollution (odour, dust, fire, expansion of toxic gases methane and carbon dioxide—trapping heat in the atmosphere), soil pollution (damage to vegetation) and natural environment (Vaverková, 2019). Importantly, 83.52 m^3^ of methane have been produced from a ton of waste in kakia open dump and in one year, 1–40 m^3^/ton of CH_4_ can be produced by 200–300 m^3^/ton of fresh MSW (Osra et al., 2021). The negative context can be turned through positive technologies like energy conversion methods, producing an electrical current, biogas (Mishra et al., 2021), etc., For example, Gaibor L., have reported the production of gas and electrical power from the sanitary landfill viz., 95.04 m^3^/min of biogas have been obtained and their net electric power is about 12134.71 in the year of 2012. Later on, from 2013-2020 the net electric powers like 13223.82, 14703.63, 116194.93, 17701.56, 19223.5, 20762.04, 22406.56, 24132.8 was calculated from 103.58, 115.16, 126.84, 138.64, 150.56, 162.61, 175.49 and 189.01 m^3^/min of biogas (Gaibor, 2021). In earlier days, open dumping method of waste disposal has been adapted by three-quarters of the countries and territories around the world (Rushbrook and Organization, 2001). The dumpsite is an important component of integrated waste management systems whereas each process has moderate danger from generation to termination like waste pickers and child scavengers in probable exposure to waste hazards. As an upgrading step limiting their access to the site can be restricted through the entrance gate and proper fencing of the surroundings. Construction waste should be used as a covering material over the waste. For effective waste delivery any truck which enters through the gate should be monitored through the electronic board recorder and Weigh Bridge. Another important measure to be followed is that regarding waste unloading placement plan, in which wastes can be dumped in specific areas or “cells” instead of spreading the waste to overall areas (EPA, US., 2002; Zamorano et al., 2009). Also, the tipping face should be accessible through developing infrastructure like roads which helps to manage waste in easy ways. Once heaping of particular cells is over it should be covered before moving to the new cells for waste dumping. Developing green buffer over the landfill sites helps to reduce bad odour and it provides better visual impact to the nearby residents (Bhawan, 2016). Peter AE, Nagendra SS., have reported the impact on PM2.5 emissions from 30-yr Open Municipal Solid Waste (OMSW) in Chennai, India. The highest concentration of 247, 136 and 53.4 μg m^−3^ of PM_2.5_ emissions have been observed using AERMOD simulate methods and the emissions have been recorded in nearby residential places (Bhawan, 2016). Importantly, Open Dumpsite (ODs) multiplication around residential sites causes severe health issues like malaria and breathing difficulties (Aluko et al., 2021).

## 3 Bio-waste management

The prospective construction of high value-added chemicals and bioenergy with a negative feedback loop is called “bio-waste”. The bio-waste has created a direct impact on the greenhouse gases by illogical use of fossil fuels (Aluko et al., 2021), the influence of population on freshwater sources and energy demand for industrialization may trigger the chances of global warming. Importantly, as a major waste management system incineration and landfill processing are used, which emits greenhouse gases that can cause global warming (Astrup et al., 2009; Kim and Kim, 2010). As per the Indian government guidelines, the sorting and segregation of waste into different specific containers for further steps of recycling and decomposing should be performed appropriately. Generally, the decomposing time of waste materials are varying from one to another. Decompose property lies on the nature of the material chemistry. For an example, monofilament fishing line, sanitary pads take 100 yr for decompose same time vegetables takes 5 days to one month for decomposition. The common waste items, decomposing time and their risk of toxicity have shown in Table 1.

**Table 1:** Common waste items decomposition time.

Waste Items	Decomposition time	Risk of Toxicity
Cigarette butts	18 months to 10 yrs	High (cadmium (Cd), lead (Pb) and arsenic (As)
Plastic bags	10-1,000 yrs	Dioxins: lethal persistent organic pollutants (POPs) and 2,3,7,8 tetrachlorodibenzo-p-dioxin (TCDD), agentorange
Tires	2000 yrs	benzene, metals such as lead, polycyclic aromatic hydrocarbons such as benzo(a)pyrene, and synthetic rubber components such as butadiene and styrene
Plastic bottles	450 yrs	dioxins, furans, mercury and polychlorinated biphenyls
Synthetic fabric	More than 100 yrs	polyester and nylon contributes to microplastic pollution
Disposable diapers	500 yrs	xylene and ethyl benzene
Batteries	100 yrs	Hydrogen, Hydrogen Sulphide, Arsenic Hydride and Antimony Hydride gases.
Clothes	6 months	nonylphenol, benzidine, formaldehyde, (heavy) metals
Leather	50 yrs	mineral salts, formaldehyde, coal-tar derivatives, and various oils, dyes, and finishes, some of them cyanide-based.
Sanitary pads and tampons	25 yrs	volatile organic compounds and phthalates

Initially, the wastes disposed in the dumpsites are manually hand-sorted, but nowadays many sorting plants were constructed for the industrial and constructional waste sorting process. For example, both inert and non-inert materials present in the construction waste are segregated on the basis of materials which helps to minimize waste from its source of origin (Yuan et al., 2013). Many industries and household wastes were recycled and disposed by the following standard guidelines. According to the US guidelines, the Environmental Protection Agency (EPA) has registered the broad spectrum of American industrial facilities for about 7.6 billion tons of industrial solid waste disposal every year (EPA Guide, 2021). The regulatory mechanism and guidelines has variations in states, tribal and small governments. In India, Bangalore has adopted specific guidelines Bruhat Bengaluru Mahanagara Palike (BBMP) for the segregation of household wastes in 2003. The wastes have been categorized into three sections 1. Organic waste (kitchen waste and garden waste), 2. Dry wasteplastic (must be rinsed if soiled), paper (must be rinsed if soiled), metal, glass, other dry waste, E-waste (should be hand over separately), Construction debris/inert, broken glass 3. Sanitary/Reject waste (sanitary waste (with proper newspaper wrapping) and sharps. Industries and hospitals were following several schemes and plants for bio waste treatments. pollution control board has started following certain biomedical waste (management and handling) rules, then after it has been upgraded to revised versions of rules which was known as BMW Management Rules, 2016 (biomedical waste segregation, treatment and disposal). According to these BMWM rules, medical wastes are categorised as non-infectious (75–85%), infectious (10–12%) and hazardous (5–10%) wastes. Medical wastes are segregated into 5 different colored plastic bags or containers 1. Black for the collection of cytotoxic drugs and chemical wastes; 2. Yellow for the collection of human and animal anatomical wastes, solid waste, expired or discarded medicines, chemical waste, micro, bio-clinical lab waste and chemical liquid waste (in non-chlorinated plastic bags). 3. Red for the collection of contaminated wastes (recyclable) such as tubing, bottles, intravenous tubes and sets, catheters, urine bags, syringes (without needles) and gloves must be collected in non-chlorinated plastic bags and containers. 4. White for the collection of waste sharps, including metals that has to be disposed in puncture, leak and tamper proof containers. 5. Blue for the collection of glasswares and that has to be kept in cardboard boxes. According to the report of Swachhata Sandesh Newsletter (2020) launched by Ministry of Housing and Urban Affairs (MoHUA), Government of India has successfully achieved 100% waste segregation at household levels in 63,204 wards (74.82%) (Singh, 2019). In general, the SWM rules are applicable for the persons who generate, collect, receive, store, transport, treat, dispose, or direct other waste including biomedical waste (BMW management) in all forms. The source segregation of waste has been commanded to drive the waste to fortune by adopting 3R rules as recovery, reuse and recycle. The SWM rules 2016 has to be followed mandatorily, or else, in future within 2 yrs solid waste processing facilities can be positioned up through all provincial bodies having 1 lakh or more people and within 5 years we must close all old and reckless dump sites.

On our earth, most often the renewable energy sources were used for many industrial and domestic purposes whereas, many researchers were looking for alternative ways for the production of energy from the wastes that provides the salient features in both environmental and economic status. The bio waste treatment technology has been categorized into 13 technological contexts such as 1. Direct use has three sections viz., direct land application for crops production, direct animal feed for meat/fish production and direct combustion for cooking fuel/heat/electricity. 2. Biological treatment has five sections—composting for crops, worms and vermicomposting for meat/fish production, black soldier fly treatment (larvae for meat/fish production) (residue for crop production), Anaerobic digestion treatment has two ways of recycling viz., digest for crops and biogas for transport fuel. 3. Physio-chemical treatment has two sections—Trans-esterification for glycerol production have used meat and fish production and as biodiesel for transport fuel and in another phase of the densification process produce pellets and briquettes used in cooking/fuel/heat/electricity. 4. Thermo-chemical treatment process provides char from pyrolysis progress used in cooking and meat/fish production, bio-oil were produced from the process of liquefaction that is used as transport fuel and the final category of thermochemical treatment is gasification that produces gas which is used for cooking fuel/heat and electricity (Lohri et al., 2017). Importantly, the potential production of high-value products such as bioplastics, biochemical and bio-based materials were generated from the source of heterogeneous bio-waste which can be obtained in a limited range. The production of bioethanol from the matooke peals is an effective inventive step for the ethanol production. After the process of segregation of house hold waste or banana industry waste, the matooke peals could be processed for the ethanol production and the production steps are matooke farming, matooke peal extraction, coarse powder of dried peels, hydrolysis, fermentation, incubation for 18 h and analysis of matooke bioethanol (Yusuf and Inambao, 2019).

### 3.1 Science of composting and its types

#### 3.1.1 Windrow composting

The windrow (long row) composting is used for the production of compost by piling the waste materials such as biodegradable and non-biodegradable for obtaining a high yield. In general, the composting process consists of three major processes such as pretreatment/material reception, composting and post-treatment process. To achieve successful composting, the materials must be initially shredded and blended, to give an optimum level of nutrient balance and moisture content for effective composting. Later, the post-treatment process was used to eliminate the large unwanted particles through screening methods. The principal content of the windrow composting process is aeration, temperature and moisture content (maintain the optimum conditions for microbial growth). Agitation, forced agitation or both mixed options can be applied in the composting process and the selection type is based on the waste material as well as the on the air requirements of the microorganism (Stentiford, 1996). For the aerated (turned) windrow composting process, large volumes of waste were more suitable (Ahuja et al., 2014). They are collected or sourced from the high-volume food-processing enterprises, industries, local governments and non-government authorities (Agarwal et al., 2015; EPA US, 2021). The pile turning and pile standing periods are parts of windrow composting process and very frequently pile turning process disturbs the microbial formation and heat up. Usually, the pile turning process are done once in 2–4 weeks of interval for getting quicker compost (EPA US, 2021). Generally, the accumulated compost carries a high quantity of N_2_O which was triggered using pile turning process showing quite higher emission than the simple gaseous emission (EPA US, 2021). Windrow composting system was also used in different waste management such as horticultural waste management, dairy manure, biodegradable Municipal Solid Waste (MSW) management and chicken manure (Ayilara et al., 2020). Collective reports reveals that the denitrifying bacteria community has been used for the Calcium Superphosphate-Mediated Reshaping process from the Pig Manure Windrow Composting (Jin et al., 2021). Greenhouse gases emitted during the windrow composting process are CO_2_, CH_4_, and N_2_O (Vergara and Silver, 2019). Industries and domestic wastes are the major sources that could cause severe environmental problems. The solid-waste quantity of Lahore (located in the capital of the Pakistani province of Punjab) per day is 5000 tones which consist of 60% biodegradable and 40% non-biodegradable waste (Ahmad et al., 2017). In this case the reduction of composing period is achieved by introducing the specific microbial activity. Naturally, in aerated windrow composting the solid waste matters are converted into organic matters with a yield of 25% organic content along with the achievement of carbon-nitrogen ratio of about 26:1 (Kerala Environement Conference, 2014). The windrow composting layout has 3 phases such as initial activation, mesophilic/maturation and thermophilic phase (Barthod et al., 2018). In the process of large scale windrow composting initially, large number of raw materials will be transferred to form a windrow formation with a long rows of flux size of about height, 4–8 feet vs. width, 14–16 feet (Barthod, 2021). The pile temperature will be evenly distributed and maintained with proper oxygen flow (windrow’s core). It takes around 2–4 months for full decomposition using Windrow composting methods and the turned products will be analyzed by a sieve analysis (gradation -particle size distribution) after that the final compost will get obtained (EPA US, 2016). For reducing the time of compost preparation, the microbial solutions can be showered in the turning process. The essential of windrows composting have focused in the warm and arid climate by covering the compost to prevent the water evaporation and in rainy seasons the shape of pile should be adjusted so that the water would runoff instead of being absorbed by the pile. The major advantage associated with windrow composting is that it involves less investment with low maintenance support but it is labor-intensive, requires more space and processing time (EPAUS,^2016^).

#### 3.1.2 In-vessel composting

Large volume of waste with limited space was in demand with in-vessel composting system rather than windrow’s composting system. Indian government mission of Swatch Bharat aim can be achieved by in-vessel composting by linking daily household waste with resource generation (Manyapu et al., 2017). All types of wastages such as organic and bio solids can be treated in this system. The in-vessel management system can be influenced in many contexts such as large volume of food wastes, organic, pig manure composting, agricultural waste, septage management of pharmaceuticals, personal care, etc. In this method, the wastes can be composed inside the building tanks, containers or vessels in which the airflow can be controlled with appropriate temperature and moisture conditions. The defined level of carbon-to-nitrogen (C/N) balance and aeration level plays a vital role in the regulation of emission (Ayilara et al., 2020) in aerated process. The aeration management system has encountered gas emissions at the rate of 0.9% and 3.8% of green gases of methane and nitrous oxide in passive mode and >90% of CO_2_ in active mode. These results indicate the merits and profitable outcome which can be achieved by an active mode of aeration process rather than the passive mode (Thomas et al., 2020). The product as a compost of in-vessel food waste composting system provides salient features in economic status like the replacement of fertilizers that provides vital and emerging nutrients for plant growth as well as 80% of respiratory effects have reduced in the food waste composting systems (Mu et al., 2017). Importantly, the profit obtained per year is $13,200 by marketing the vegetables which was grown by this compost (Mu et al., 2017). This system is quietly complicated, as it requires proper expertise for the operation and handling. The in-vessel co-composting management system also plays a vital role in different versions of wastages produced by humans like household (Iyengar and Bhave, 2006). For septage human waste management, the in-vessel composting system has been considered as an effective method. Importantly, 44% of Indians depends on the septic tank human waste management systems. In-vessel co-composting is considered as an option for resource recovery from septage treatment (Thomas et al., 2020).

#### 3.1.3 Recent context of vermi-composting

Our planet has more than 3000 different species of earthworms in different parts of the world (Zicsi, 1982). Vermicomposting is nothing but the production of compost through decomposition process by utilizing different species of earthworm or production of humus like particles obtained by the conversion of organic materials. The compost consists of rich nutrients such as carbon, nitrogen, phosphorous, potassium, magnesium and calcium as well as it enhances the physical, chemical and biological properties of the soil. The Compost will improve the properties of the soil such as electro conductivity, pH, aeration, penetrability, bulk density, soluble salts and water preservation. Mostly, the temperate species of earthworms are used for composting like *Eisenia fetida* (Tripathi and Bhardwaj, 2004), *Lumbricus rubellus* (Cholilie et al., 2019), *Perionyx excavatus* (Parthasarathi et al., 2016) and *Eudrilus eugeniae* (Balachandar et al., 2020). The composition of vermicomposting by the polyculture and monoculture of *E. eugeniae*, *E. fetida*, and *P. excavatus* using sawdust simultaneously with press mud (Dominguez et al., 1997). The improvement of decomposition serves to enhance the phosphorus content of vermicompost and the decomposition of organic waste was increased excessively through earthworm remarkably with enhanced product quality. Enzymes and microbial communities within the earthworm are the basis for efficient production (Subramanian et al., 2010; Zhou et al., 2019). The compost can act as an effective organic fertilizer for farming which helps to enhance the yield naturally. Vermicomposting act as an effective alternative treatment method for major waste management systems for organic waste in an economic and environmental friendly approach. Importantly, the value-added horticultural plant growth medium was reported to have 10–20% of higher yield and possess higher commercial value in the market. In recent times, many advance technologies have been incorporated with vermicomposting to ameliorate the economic values of their production. Engineers from the National Institute for Agricultural Engineering (Silsoe, England) have constructed the device which has mechanically driven the large containers used for adding the waste at the upper region and collection of compost when the waste passes through the mess (agitation and filtration). The hydraulically-driven continuous flow reactors are able to handle 3 feet of organic waste in a fully automated manner way in a batch reactor (Dominguez et al., 1997). In an economic view, using this system it is possible to achieve $15,000–$30,000 from 1000 tons of waste per year and in total 27% of waste can be managed using this system.

#### 3.1.4 Black soldier fly processing

The Black Soldier Fly (BSF) is a novel approach to deal with the biowaste by insect larvae (*Hermetia illucens*). In principle, the BSF larvae have been used in the potential bio-waste conversion process by simulation of mid gut digestion to obtain high value-added products (Rindhe et al., 2019). Waste treated larvae contain ±35% protein and ±30% crude fat that could be used as an effective alternative as fishmeal as well as it can be used for fish and chicken farmers. Another important aspect of BSF larva is that it reduces the bacterial load of *Salmonella spp* for up to 80% and thereby reducing the risk of disease transmission (Rindhe et al., 2019). It is a cost-effective method which requires only lower budget but effective in reducing the waste level up to 80% which helps to minimize the usage of landfilling and open dumping process. High frequency of nutrients, organic matters were carried by residues of the larvae and could be used for farming while reducing the soil depletion too. In an economic perspective, the production of biomass from high waste is about 25%. The BSF treatment system has 5 processing facility such as 1. BSF rearing unit (it has two separate units one is for breeding maintenance colonies in captivity and the second one is about larvae growth), 2. Waste receiving and pre-processing unit (collected wastes processed mechanically), 3. BSF waste treatment unit (Rearing process), 4. Product harvesting unit (end life cycle of the product), 5. post-treatment unit (larvae refining and residue processing). The refined larvae feed can be used as an animal feed such as fish and chicken while processed residue can be sold to the market.

### 3.2 Anaerobic digestion technologies

The chemical breakdown of a substance (fermentation) under the condition of lack of oxygen (anaerobic) is called anaerobic digestion that process carried out by anaerobic microbes. Generally, the process was used for the energy production from the domestic and industrial waste. The anaerobic digestion process is 100% renewable and their energy consumption for the process have been taken from the solar power system (Energy, 2019). The process of anaerobic digestion has three stages such as hydrolysis, acidification and methanogenesis (Sikora et al., 2017). The efficacy of the process depends on the level of enzymatic reactions of microbial activities in the fermentation process. The enzymes are considered as a key to the anaerobic digestion process as well as the electron transfer reactions and has an equal contribution in the production and recovering of carbon as energy (biogas) like methane (Ferdeș et al., 2020). The waste materials will be transformed into methane and carbon dioxide in anaerobic digestion and it can be enhanced by special pathways (Li et al., 2019). General steps of anaerobic digestion include hydrolysis, fermentation, acetogenesis, and methanogenesis (Sangeetha et al., 2020; Wang et al., 2018). For Food waste (FQ) various stages of anaerobic digestion (Wang et al., 2018) are there, initially, in aerobic digestion process, the collected biomass of animal waste, plant waste, slurry and manure, food and amenity wastes were transferred into the sealed container (absence of oxygen) or biogas reactor. Next, the hydrolysis process was enhanced by the additional pre-treatments of waste by electrical, thermal, biological and oxidative systems (Anukam et al., 2019) to produce protein, carbohydrates and lipids (Morales-Polo et al., 2018). Then, the acidification process was improved by adding supplementary additives which stimulates the conversion of complex cellular materials into acetic acid, propionic acid and hydrogen molecule by acetogenic bacterium (Li et al., 2019; Wang et al., 2018). Then the final stage involves the methanogenesis step which can be enhanced by the stimulation of microbial activity which influences the acceleration of direct species electron transfer that is involved in the enrichment of methane and carbon dioxide by methanogenic bacterium (Li et al., 2019; Wang et al., 2018). Eylem Doğan and Göksel N. Demirer et al. have reportedthe production of Volatile Fatty Acid from the fraction of solid waste using acidogenesis of anaerobic digestion (Doğan and Demirer, 2009).

### 3.3 Anaerobic fixed dome digester

Anaerobic digestion is the production of biogas (a mixture of methane (CH_4_), carbon dioxide (CO_2_), hydrogen (H_2_), nitrogen (N_2_), and hydrogen sulphide (H_2_S) and bio solids using anaerobic microorganism (Beychok, 1967; Nasir et al., 2012). According to the report of Environment and Infrastructure GTZ for the future worldwide have discuss the steps and process of anaerobic dome digester viz., the fixed dome plant is an immovable closed rigid circuit called compensation tank. Initially, the waste materials can be filled in the mixing tank through the inlet pipe and sand trap. The self-agitation process was handled by biogas pressure in a sudden blast that helps in mixing the reactor content. The slurry will be relocated to the compensation tank once the gas production begins. Generally, the plant is located in the underground so that it is not influenced by physical damage as well as space occupy. Similar circumstances have influenced the anaerobic bacteriological processes. The gasholder which is present in the upper region of the digester was adjusted to collect the reserve gas using the gas pipeline. The accumulated thick sludge can be allowed to exit by an outlet pipe. The gas storage of fixed dome is about 20 m^3^, gas pressure is 60–120 mbar, and time durability of unit is less than 20 yrs whenever the methane emission is high. The defined biogas engineers can only construct the plants but the manpower cost is affordable. The material demand is always there for processing and this system provides a high yield of biogas like methane, carbon dioxide and nitrogen (Biogas, 2021). Uche AM et al. have reported that 56.4% of methane gas, 35% of CO_2_ and 6.9% of nitrogen was produced from cow dung and water hyacinth while the biogas has fixed in their staff canteen (Uche et al., 2020).

### 3.4 Biogas formation

Biogas production is one of the best method to generate energy from organic waste materials such as food scraps and animal waste under the anaerobic condition (Schwarz, 2007). Generally, the decomposition process has found to be provoked under the anaerobic condition. The fossil fuel energy demand and the frequent release of greenhouse gas levels are the major issues of the environment which has to be sorted out (Cherubini et al., 2008). The biogas formation has to cross four stages to reach the yield with the same theme of anaerobic digestion but with variations in the versions and plants. The insoluble organic polymers were breakdown in the process of hydrolysis which aids the acidogenesis step (conversion of sugars and amino acids into hydrogen, ammonia and carbon dioxide). Then, the conversation process will be carry out by methanogenesis (conversation of acetic acid, ammonia, hydrogen, and carbon dioxide to Methane and Carbon-dioxide) (Sherman). In recent studies, biogas formation has been upgraded by increasing the production value using different strategies such as particle size reduction of wheat straw, foam formation, stabilizing the divalent iron and sucrose by pectin, pretreatment of oxidation system in wastewater, proper agitation and over acetogenesis reactions (Moeller and Zehnsdorf, 2016). In earlier stages, an enormous volume of bio-waste has been collected and pre-treated using ultrasonic treatment, alkaline treatment, oxidative treatment, microwave irradiation, thermal treatment, thermochemical and sono-thermal treatments (Karuppiah and Azariah, 2019).

### 3.5 Slow pyrolysis

The charcoal-like substance generated from the burning of an organic material is called pyrolysis (Baskar et al., 2019). It’s an effective system to save the carbon, for an example, reports have revealed that 30% of energy can be produced from multi-stage pyrolysis (fixed-carbon yield of 25.73%) (Oyedun et al., 2012). It is an anaerobic process (Ghosh et al., 2020) as well as improve the soil fertility, potentially enhancing revegetation of soil regulate the ratio of contamination (Vidonish et al., 2016). Generally, a stable form of carbon cannot easily get away into the atmosphere because it is thermodynamically most stable (Martinez-Canales et al., 2012) and notably, bio-char converts the carbon into a stable form along with energy and heat production during pyrolysis that can be considered as a form of clean energy. This system creates a solid form of carbon to create fuel, graphene can be used in various sectors (anti-corrosion coatings, precise sensors, provoke the speed of electronics, solar plants, filters, electric car batteries, DNA sequencing and drug delivery) and industrial processing and proceedings. The slow heating pyrolysis can be carried out in pyrolysis bioreactor either as batch type and/or fixed bed reactor and the parameters such as temperature, heating rate, size and amount of the input material can be varied for better optimization (Vieira et al., 2020). An effective bioenergy production has been obtained from pyrolysis of Ugandan biomass wastes. The biomass has been prepared from matooke peals and it was processed for the electricity and bioethanol production. Importantly, the results of biomass analyses have revealed the presence of high amount of cellulose and Zinc (Yusuf and Inambao, 2020). The cellulose, hemicellulose and lignin was eliminated from matooke peals during the process of pyrolysis. The obtained bio mass contains more amount of volatile matter which in turn confirms the progress of carbonization which helps in combustion process. These indications reveal the possible production of bio fuel and photovoltaic from this UM-WP biomass (untreated Mbwazirume waste peel) (Yusuf and Inambao, 2020).

### 3.6 Char, charcoal, biochar

Char, charcoal, and bio-char are the different forms of carbon which possess a wide array of technological advancements in material sciences and soil management. Char, manufactured by subjecting coal to a range of mechanical pressures (Matsuoka et al., 2005) (occurs at temperatures up to 600 °C by slow pyrolysis) and vitrinite content employing pyrolysis which has a higher structural similarity to charcoal (Salaudeen et al., 2019). Yet bio-char, obtained by heating organic matter from natural sources in oxygen hindered environments, was preferable for bio-waste management (Wang et al., 2018). Bio-char, with its charcoal-like properties, further helps in alleviating climate change and enhances the overall soil quality in the biosphere (Kumar et al., 2021a, Kumar et al., 2021b) It is profoundly porous, weightless, fine-grained compound also contains nitrogen, oxygen, and hydrogen other than carbon (Rawat et al., 2019) in its physical structure. Bio-char has its origin from the 2000-yr-old practice of the Amazons, to convert their barren soil into habitable terra preta soil (Rawat et al., 2019) in its physical structure. The bio-char consists of 70% carbon and the remaining elements are hydrogen, nitrogen and oxygen (Biochar, 2018). Recent reports reveal that the current usage of bio-char has reached its peak in the bio-waste management sector and it has a wide array of raw materials to choose from, nutshells to chicken litter based on their availability. On heating, bio-char accelerates its mesoporous and microporous characteristics at different temperatures, specifically up to 450 and 550 °C (Sakhiya et al., 2020).

### 3.7 Collection, segregation and various treatments of municipal solid waste

**Figure 3:**
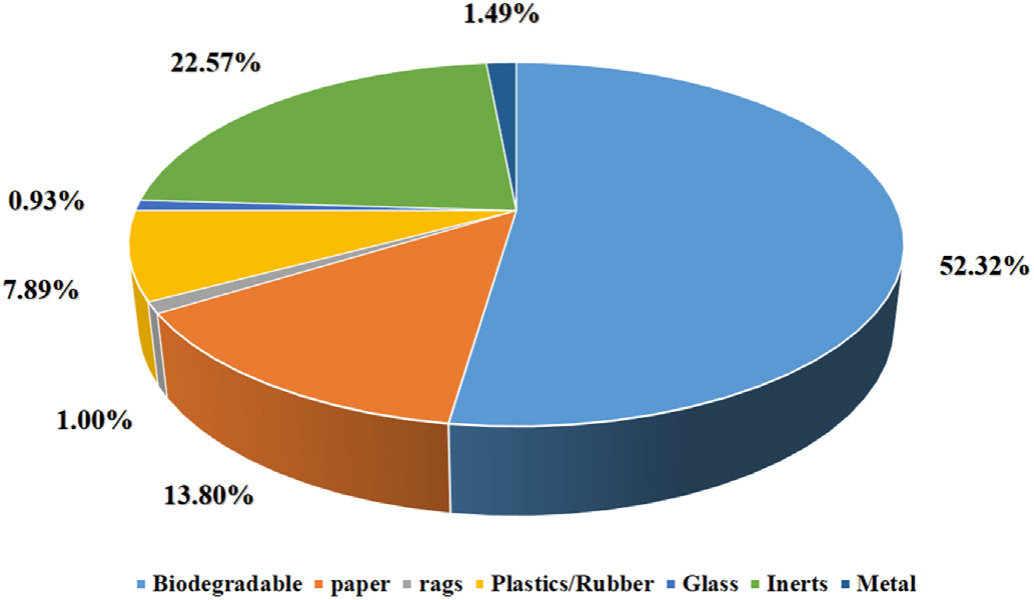
Pie chart of composition of municipal solid waste that are collected in India. According to recent report of 2019, the biodegradable waste (52.32%) is the major source that are collected followed by inert (22.57%), paper (13.80%), plastics/rubbers (7.89%), Rags (1%) and glass wastes (0.93%).

In recent times, many advanced technologies were upgraded for the collection of waste materials. Smart waste bins and trash cans have been used for the collection of waste materials as well as for the projection of toxic gas levels and fill levels (Dugdhe et al., 2016). Singh A et al., have discussed about the different waste collection systems, their demerits and the solutions to overcome it in the Indian context and suggested to adopt the usage of Infrared sensors for the IoT based waste collection systems in India (A. Singh et al., 2016). The Indian city Vellore has chosen for the discussion of possible collection methods for solid waste management in India. He also suggested that the Geographic Information System (GIS) will be one of the best method for the collection of wastes, estimation of vegetation land cover and transport optimization of waste materials (Lella et al., 2017). After the collection of municipal solid wastes, it will be segregated by various methods like grey level aura matrix, (Hannan et al., 2012), Deep learning algorithm in artificial Intelligence (Hannan et al., 2012), Arduino in scrap industry (Rafeeq and Alam, 2016), Convolutional Neural Network (Azis et al., 2020; Thanawala et al., 2020). The major source residual waste is biodegradable waste (32.5%) that is followed by miscellaneous (24.9%), paper and card (14.6%), plastics (18.8%), Recycle glass (2.5%) textile (4.7%), metal (0.3%) and metal packing (1.8%) (BrustiBin, 2016). The sources of municipal solid waste materials report for India for the year of 2019 have shown in Figure 3. In worldwide, only 13.5% of waste materials have undergone the recycling process. The disposal treatments like incineration and composting represents only 11% and 5.5%. The controlled landfill represents only 4% but sanitary landfill, landfill (unspecified) and open dump are the major concern with 7.7%, 25% and 33% (Kaza et al., 2018). Other kinds of disposal account for less than 1%. Generally, Internal Factor Evaluation and External Factor Evaluation matrices have been used for the identification of Strategic Factors of Household Solid Waste Segregation (Ghanbari et al., 2015). In most cases, the collected materials will be processed under waste treatment plants for proper disposal. The global trend in waste treatment and disposal was followed as reported in the year 2018. The segregated waste materials will be processed under various methods of treatment system for elimination and production of energy from waste materials (recycling). Waste treatment methods like incineration (Nabavi-Pelesaraei et al., 2017), compositing (Diaz et al., 2020), controlled landfill (Mavakala et al., 2016), sanitary landfill (Mavakala et al., 2016), unspecified landfill (Kaza et al., 2018), open dump (Muenmee et al., 2015) and recycling systems (Malinauskaite et al., 2017) are used as solid waste managing systems and these methods are given in Figure 5. Significantly, incineration is one of the best waste treatment processes which involves the combustion of substances in waste materials and the massive residues are collected as ash, flue gas and heat. The types of incineration techniques are rotary kiln, fluidized bed, liquid injection, multiple hearths, catalytic combustion, waste-gas flare and direct-flame (Cheng et al., 2020) method. This technique can be used for varieties of waste like medical waste, E-waste, bio waste etc. Incineration has several advantages to reduce the quantity of waste, production of heat and power, production of methane gas and ejection of the harmful germs and chemicals. Additionally, incineration process not only recover the energy from burning the waste, it also reduces the solid waste volume by almost 90% and thus it significantly provides a deviation from disposal of waste through landfilling method. The worldwide scenario of waste disposal and treatment have shown in Table 2. Based on their economic and environment status of worldwide countries have following their most preferable waste disposal and treatment options viz., East Asia and Pacific country (46%) preferable waste disposal and treatment option is landfill (unspecified), Europe and Central Asia (25.6%), Middle East & North Africa (52.7%), South Asia (75%), Sub-Saharan Africa (69%) preferable waste disposal and treatment option is open dump, Latin America & Caribbean (52%), North America (54.36%) preferable waste disposal and treatment option is sanitary landfill.

**Figure 4:**
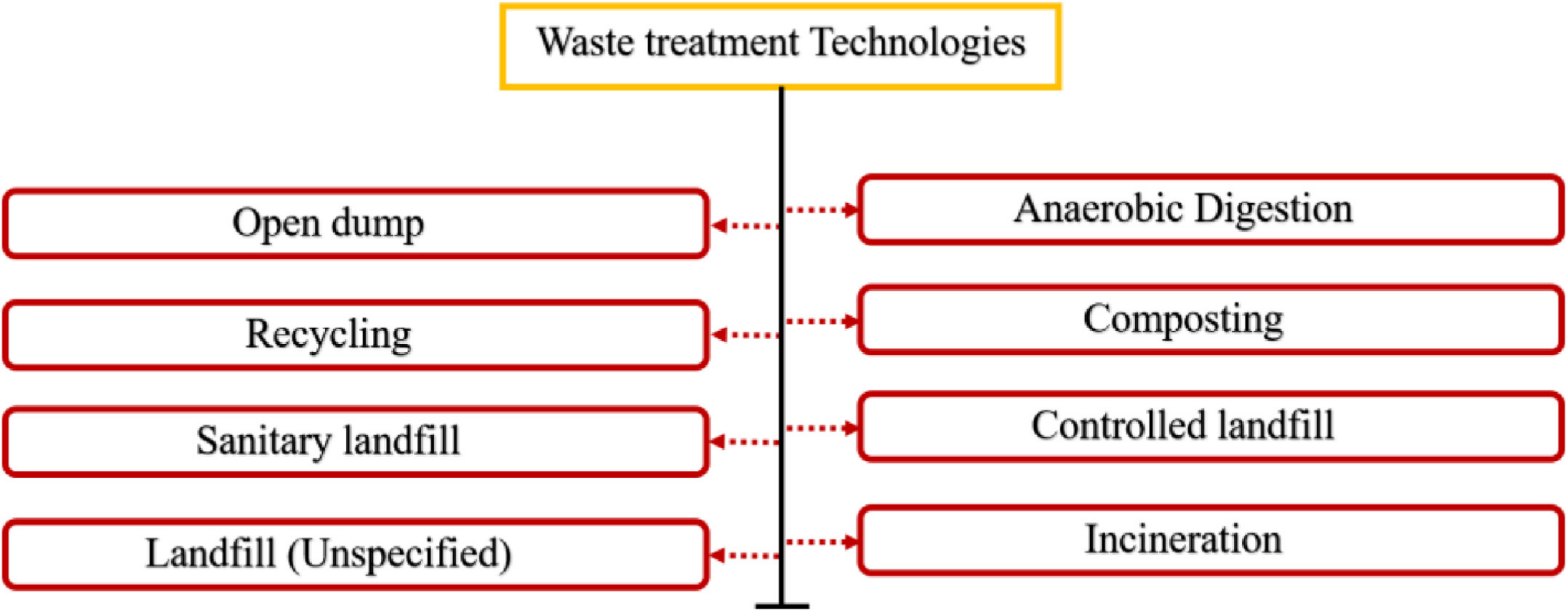
Waste treatment Technologies. Most preferable options for waste disposal treatment technologies are open dump, recycling, sanitary landfill, landfill (unspecified), anaerobic digestion, composting, controlled landfill, incineration.

**Figure 5:**
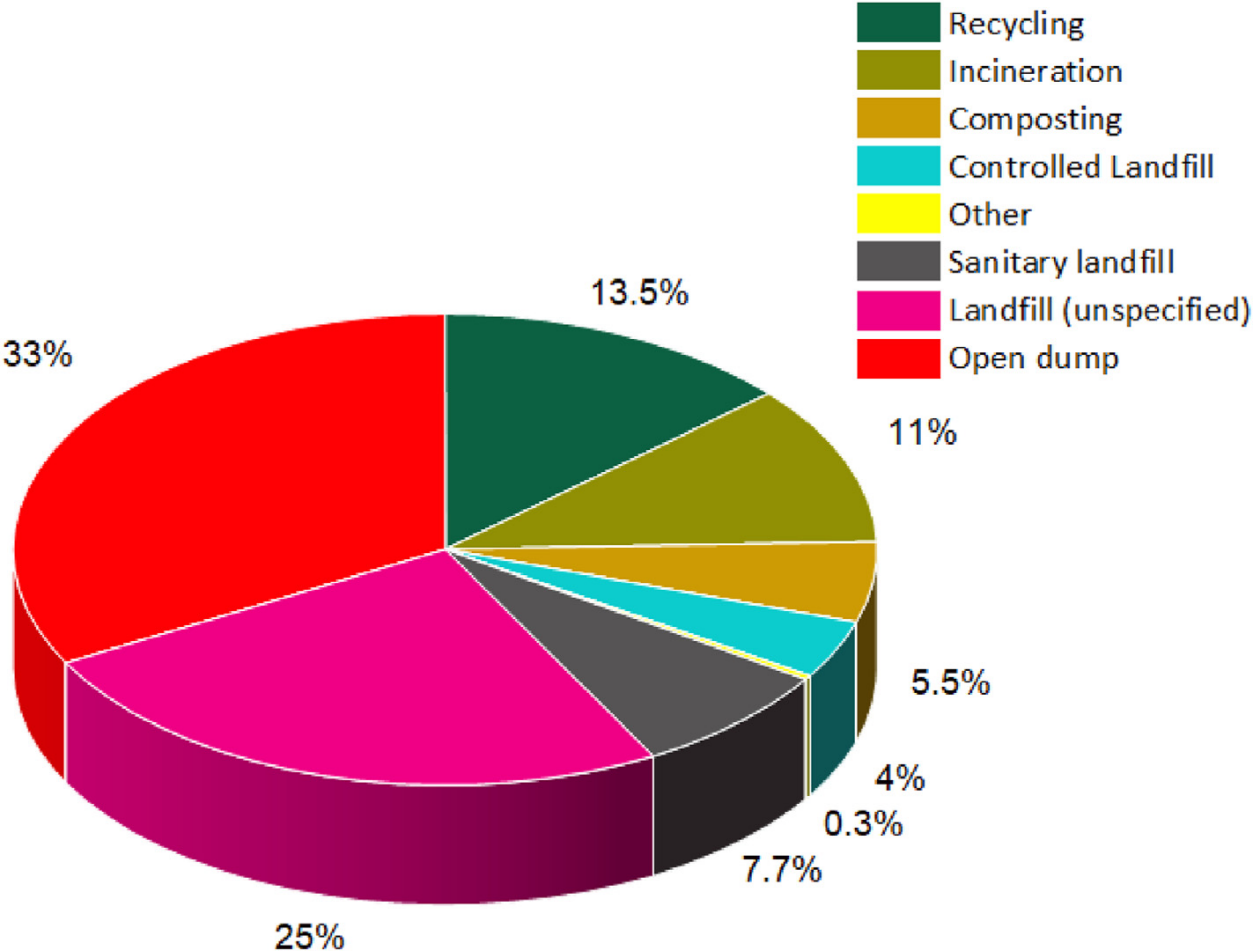
Global trend in waste treatment and disposal followed as reported on year 2018. Worldwide only 13.5% of wastes only undergone recycling process. The treatment like incineration and composting represents 11% and 5.5%. The controlled landfill represents only 4% but sanitary landfill, landfill (unspecified) and open dump are the major concern with 7.7%, 25% and 33%. Other kind of disposal accounts for less than 1%.

**Table 2:** Worldwide Scenario of Waste disposal and Treatment.

	Anaerobic digestion (%)	Composting (%)	Controlled Landfill (%)	Incineration (%)	Landfill (unspecified) (%)	Open dump (%)	Recycling (%)	Sanitary landfill (%)
East Asia and Pacific	0	2	<1	24	46	18	9	<1
Europe and Central Asia	0	10.7	1.3	17.8	20.1	25.6	20	4.5
Latin America & Caribbean	0	0	15	0	1.5	26.8	4.5	52
Middle East & North Africa	0	4	14	<1	9	52.7	9	11
North America	0	0.4	0	12	0	0	33.3	54.3
South Asia	0	16	0	0	4	75	5	0
Sub-Saharan Africa	0	<1	11	0	12	69	6.6	1

### 3.8 Conversion of waste to useful energy

The Waste-to-energy (WTE) conversion offers an excellent alternative to fossil fuel combustion. Municipal solid waste (MSW), burns practically cleaner than many fossil fuels. The energy conversion from the waste to generate electricity by burning solid waste found in the landfills is a vital treatment process. It helps in reduction of waste in landfill sites, reduction of carbon emissions and reduction of the use of fossil fuels. The energy can be recovered from the biodegradable and non-biodegradable wastes by Thermo-chemical conversion (Incineration and Pyrolysis/Gasification) and Bio-chemical (Anaerobic Digestion) conversion. Electricity and heat can be generated from waste which provide an alternative and more environment-friendly source of energy. Energy recovery from MSW is gaining momentum in some populated countries of the world such as Indonesia (amelioration of binding specifications for chemicals in recycling products (Lahl and Zeschmar-Lahl, 2018), Brazil (improving the reinforces the inherent potential of MSW (Lino and Ismail, 2018), Pakistan (Circulating Fluid Bed combustion), Nigeria (landfill gas recovery and anaerobic digestion), Bangladesh (combustion, gasification, pyrolysis, composting, plasma arc, refused derived fuel), and Russia (separation of commercial and institutional waste) (Mukherjee et al., 2020)] as sustainable waste management alternatives.

### 3.9 Controlled disposal and resource management

Nowadays, landfill is the popularly used method of waste disposal. However, the inappropriate disposal of construction and demolition waste was recognized as a major problem around the world. But direct disposal can cause serious environmental effects and other issues. Still, scientific waste disposal processes can slightly reduce the problems and provide the waste as resource and it further leads to economic and environmental benefits (Domínguez et al., 2016). Government policies and laws remain significant drivers for the all types of controlled disposal of waste.

### 3.10 Government policy and act

**Figure 6:**
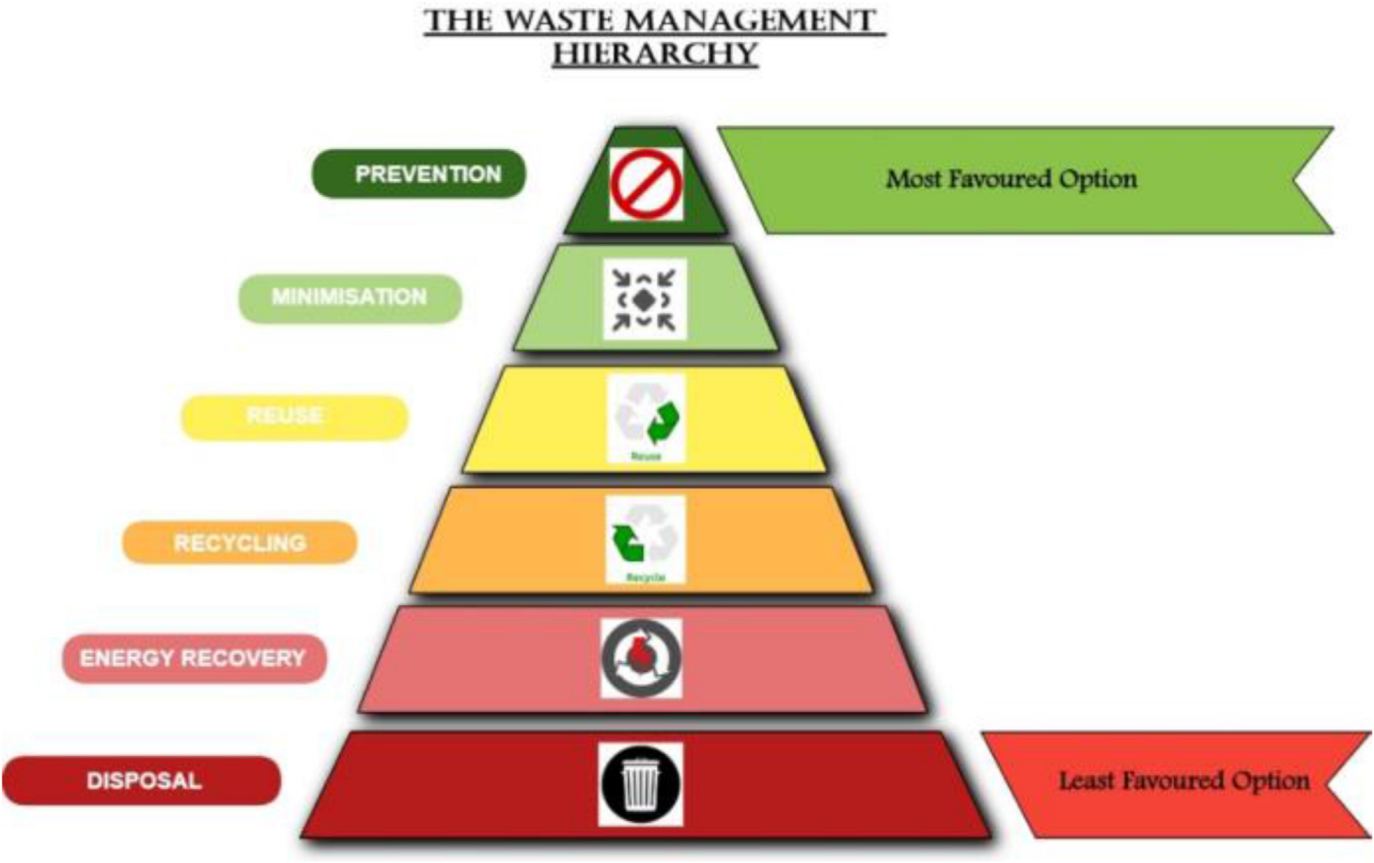
Triangular diagram of Waste Management Hierarchy. The order of preference for waste reduction and effective management are illustrated in the upward triangular diagram.

**Figure 7:**
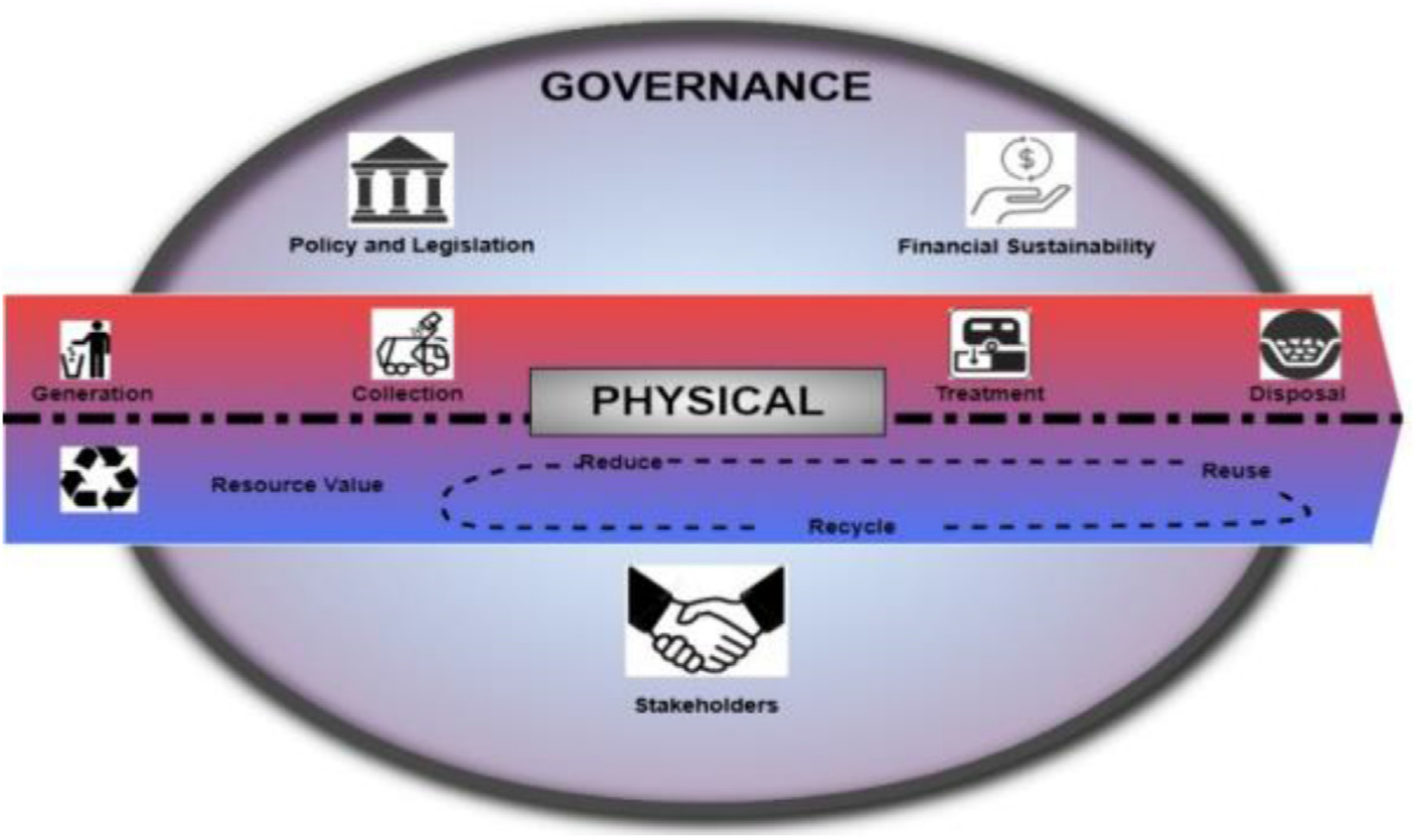
Overview of Waste management. Waste generation, proper collection, effective treatment, looking for recycling opportunities and effective disposal that are bound by government policy and legislation are the key steps in the effective management of waste.

Several countries around the world have their own policies and acts to promote a safer environment and health through effective, efficient and responsible waste management practices. Mainly the policies will be formulated to increase the less waste generation, full resource recovery, clean environment and carbon-neutral waste sector. A global agreement (The Basel Convention is an International Environmental Agreement (IEA) regulating movements was made in 1992 to handle the hazardous wastes, including WEEE (Shittu et al., 2020). This agreement prohibiting the export of hazardous waste from OECD to non-OECD countries, is still to come in force. European Union’s made legislation on Waste from Electrical and Electronic Equipment (WEEE) and it came into action in August of 2004, and that makes it mandatory on manufacturers and importers in EU states to take back their E-products from consumers and ensure environmentally sound disposal (EPA, US., 2002). Similarly, India has its own law and regulation including environmental policy and it is formulated newer environmental laws that is E-waste 2016. Union Ministry of Environment, Forests and Climate Change (MoEF & CC) has introduced the new Solid Waste Management Rules (SWM), 2016 and these management rules are applicable for plastic, e-waste, biomedical, hazardous and construction and demolition waste to emphasize promotion of waste to energy plants. Likewise, many countries including China (The Law of the People’s Republic of China (Jiaru, 2015)), Japan (The Sewage Disposal Law along with waste cleaning act, new packaging recycling law, The Waste Disposal and Public Cleansing Law etc. (Sakai, 1996), have implemented law and regulations). Generally, the waste management cycle has several stages such as waste generation from various resources, artificial intelligence, manual methods of proper waste collection from the bins and trashes, proper segregations and defined treatments like incineration, dumping, recycling opportunities and effective disposal that are bound by government policy and legislation are the key steps in the effective management of waste (Figure 6). The order of preference for waste reduction and effective management are illustrated in the upward triangular diagram. Importantly, the waste hierarchy tool was used for the production of energy from the environmental pollutants as well as to protect the environment from different pollutants. In the case of waste management systems considering the disposal option is least favoured option and prevention is the most preferred option for the waste management. This system contains six approaches for facing the burden of solid waste materials such as prevention, minimization, reuse, recycling, energy recover and disposal (Iyer, 2017) as shown in Figure 7.

### 3.11 Action plan and strategic plan

Extended Producer Responsibility is an enforced plan of environmental protection strategy which increases the responsibility of the manufacturer for the entire use of the product, recycling and final disposal of the product. The e-product producer's responsibility is protracted to the post-consumer stage of a product. Another strategic plan is that the consumers should pay the increasing tax while purchasing the electronic device which cover the future cost of recycling. Tax credits are distributed to consumers who bring their electronic waste to be recycled and to agencies that collects electronic waste for recycling. This option is probably encouraged in the e-waste recycling market and may reduce the e-waste accumulation. Consumers need to pay a fee on purchase of an electronic device, in which the certain percentage of money is reimbursed when they return the product to a certified recycler. This option provides end-of-life incentives for consumers to recycle their electronics. Consistent improvising the policies and increased social awareness may create the better environment by managing the waste.

### 3.12 Waste management—countries investment

**Figure 8:**
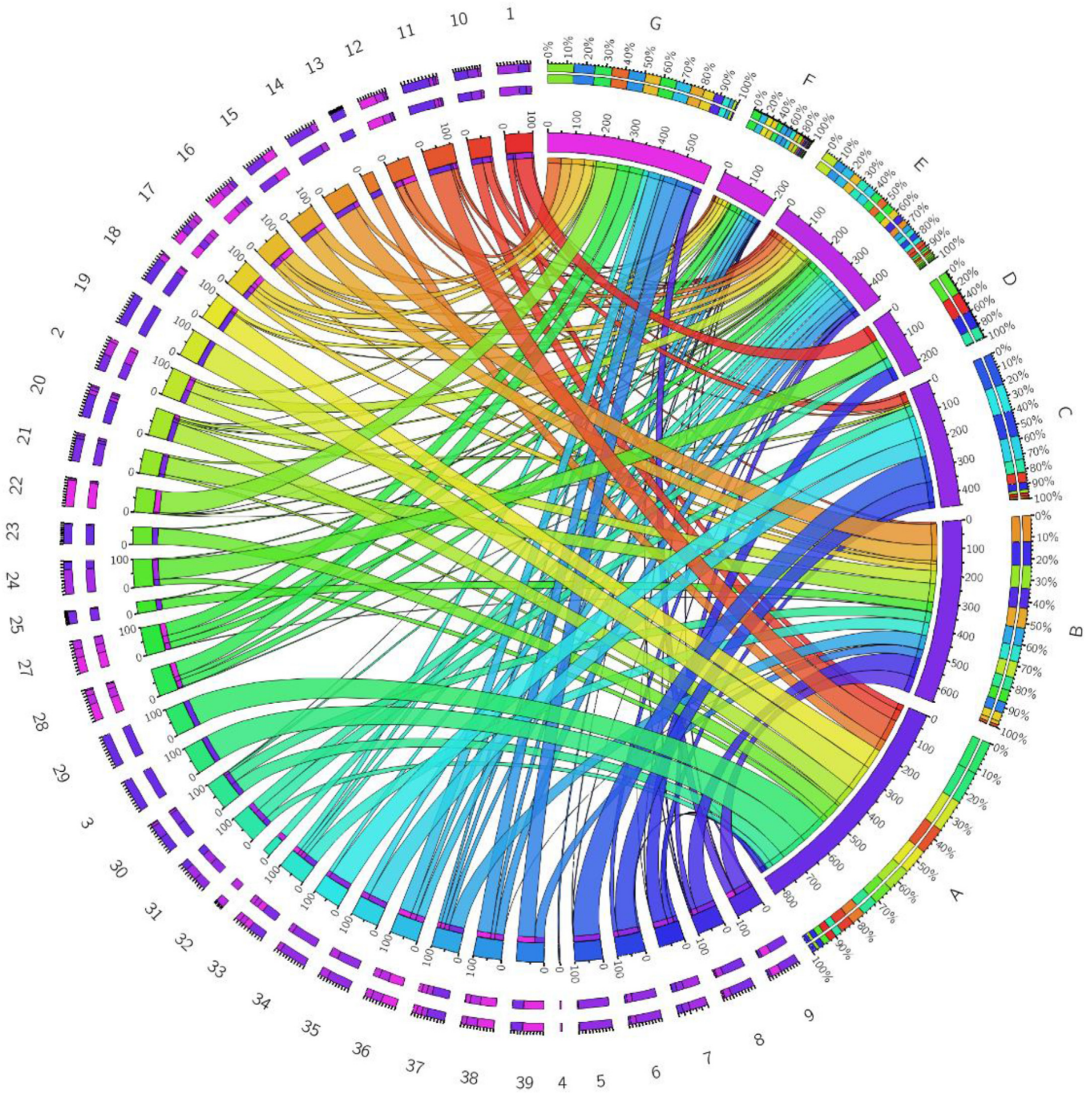
Circular plot chart for various waste management techniques and their investment. Out of 100% investment that spend for different waste management methods, Japan spends 80% of their money for incineration process Australia spends 42.1% of their money for recycling process, 100% of money for open dump was spend by Azerbaijan, Iraq and Oman, 74.5% of money for sanitary landfill was spend Mexico, 27.1% for composting in Netherland, 98% of investment in Bhutan is to control landfill and unspecified landfill is about 80% of invest in Greece. In the circus plot the alphabets A to G represents, Open dump, landfill unspecified, Control landfill, Sanitary landfill, Recycling, Composting and Incineration. The numerical numbers 1 to 39 indicate the countries viz., Argentina, Australia, Azerbaijan, Bangladesh, Bhutan, Belarus, Brazil, Canada, China, Cuba, Egypt, Estonia, Ethiopia, Greece, Greenland, Finland, France, India, Iraq, Iran, Indonesia, Japan, Kazakhstan, Mexico, Nepal, Myanmar, Netherland, Norway, Oman, Pakistan, Paraguay, Philippines, Portugal, Saudi Arabia, South Africa, Switzerland, Spain, Sweden and Tai. The corresponding waves in circus plot represents the different countries investment on different treatment methods on solid waste.

One of the biggest burdens of all countries in the world is to produce the perfect waste management system to control the solid waste. Generally, the management system was categorized into incineration, recycling, open dump, sanitary landfill, composting, control landfill and unspecified landfill. Many countries are investing a percentage of money for waste management. The method of management system may be varied depending on the country’s waste resources. Money investment on a particular waste treatment process by different countries based on their amount of waste was shown in Figure 8. It was observed that, maximum amount of money has been invested on incineration process in Japan; and the least amount of money has been invested in composting process in Netherland. In overall, out of 100% investment that spend for waste management, around 80% of money are invested in the incineration process in Japan; 42.1% for recycling in Australia, 100% for open dump in Azerbaijan, Iraq and Oman, 74.5% for sanitary landfill in Mexico, 27.1% for composting in Netherland, 98% of investment in Bhutan is to control landfill and unspecified landfill is about 80% of invest in Greece.

## 4 Awareness of waste management during covid-19

### 4.1 Covid-19 impact on medical waste

The worldwide scenario of Covid-19 is 188 million and 31 million cases have been confirmed in India till 15^th^ July 2021 (WHO n.d.). Worldwide bio-medical waste management system is highly affected by Covid-19. Unusual amount of medical waste has been recorded viz., China has reported six-fold of medical waste (increased into 240 metric tonnes) (Tassakka et al., 2020). Importantly, in Barcelona the level of medical waste has reached up to 350% viz., normal level of medical waste generation is about 275 tonnes but after pandemic its reaches 1200 tonnes (ACR, 2020). Governments have a special concern on managing the waste with new systems as well as releasing schemes and guidelines to maintain the pandemic situation properly. The Central Pollution Control Board (CPCB) has revealed the guideline (revision-4) information about handling, treatment and disposal of waste generated during treatment/diagnosis/quarantine of Covid-19 patients for controlling and managing the pandemic effectively (CPCB, 2021).

The climate change is global emergence that was expected to change the world for present and future generations. The COVID-19 crisis in 2020 made a turning point in realization for the progress on climate change. The efforts made to control COVID-19 transmission have reduced the economic activity and it led to temporary improvements in air quality in some places and the daily global CO_2_ emissions decreased by –17% compared with the 2019 levels (Le Quéré et al., 2020). This year, global greenhouse gas (GHG) emissions will fall from the previous years of record. However, it is a short time event, and after the end of the severances, the emissions rapidly increase (revenge emission) to even higher amounts than the pre covid-19 levels. On other hand the situation leads to fall of oil price, it affected the renewable energy market but not completely. However, there is no convincing evidence that either weather or climate have a strong influence on transmission (Downtoearth, 2021).

As per the present knowledge on COVID-19 and existing practices in management of infectious waste and other contagious diseases like HIV, H1N1, etc specific guidelines have been made by the MoHFW, WHO, CDC, ICM and other concerned agencies (CPCB, 2020). India has generated 18,000 tonnes of medical waste in four months, June: 3,025.41 tonnes, July: 4,253.46, August: 5,238.45 and September: 5,490 tonnes (Kapoor et al., 2020). The 3.4 kg of healthcare waste per day is generated by a Covid-19 patient and the healthcare waste is gradually increased at the peak period of Covid-19. Example, 600% percentage from 40 to 240 t of healthcare waste was increased in Hubei and other countries also facing the same challenges. During the pandemic times, the high chances of water will get contaminate by the landfill containing healthcare solid waste and cost of health care also will get increased (Das et al., 2021). Particularly, European government have facing the challenges related to maintain the household waste produced by Covid-19 patients at home, care about health care workers and make sure their safety and making more space for wastages.

In India, Central Pollution Control Board (CPCB) made guideline for Handling, Treatment, and Disposal of Waste generated during Treatment or Diagnosis and Quarantine of COVID-19 Patients (CPCB-BMW, 2016). It explains the implementation procedure to COVID-19 Isolation wards, Sample Collection Centers and Laboratories for COVID-19 suspected patients, Responsibilities of persons operating Quarantine Camps/Homes or Home-Care facilities, Duties of Common Biomedical Waste Treatment Facility (CBWTF) and Duties of Urban Local Bodies. This guideline ensures proper collection and disposal of biomedical waste as per BMW Rules, 2016. Some of the operators of CBWTF should confirm regular sanitization of workers and provided with adequate PPEs including three-layer masks, splash proof aprons/gowns, nitrile gloves, gum boots and safety goggles. It ensures to use dedicated vehicle to collect COVID-19 ward waste (Siming and Ok, 2020). It is not necessary to place separate label on such vehicles; Vehicle should be sanitized with sodium hypochlorite or any appropriate chemical disinfectant after every trip. Separate record for collection, treatment and disposal of COVID-19 waste should be maintained by CBWTF operator. Then the urban Local Bodies are responsible for ensuring safe collection and disposal of biomedical waste form Quarantine Camps or Quarantine Homes or even Home Care for COVID-19 suspected persons (CPCB, 2020) (CPCB-BMW, 2016). Reverse logistics network design has been used for the Covid-19 waste management. In brief, the medical wastages are initially collected from the existing treatment centre. The materials are handled and packed with care under the guidelines of BMW Management Rules, 2016 and CPCB BMWM guidelines for COVID, 2020. In next stage, materials are stored temporarily in Temporary treatment centres and then special landfill method have been used for disposal (Kargar et al., 2020).

### 4.2 Industrial waste and environment

Manufacturing waste, chemical waste, mining waste, oil & gas waste, nuclear waste, power plant waste, and other wastes from different industries are considered as industrial wastes. Appropriate industrial waste management system is implemented to achieve considerable economic as well as environmental benefits (Moo-Young, 2019). The COVID-19 has negatively influenced in industrial waste because the 40% of medical waste was increased due to the usage of medical products during COVID-19. On the other hand, manufacturing activity of many industries has fallen drastically due to COVID-19 scenario that highly reduce the waste production. Whereas, from the pharmaceutical and medical sectors were producing hazardous wastages in higher production. Importantly, existing hazardous waste treatment capacity was overwhelmed in developing countries due to this pandemic. The municipal waste volumes are enormously increased and effectually overwhelming the existing waste collection and disposal systems. Substantially, the plastic and other products recycling progress got slowdown and due to that reduction turn to become burden in solid waste collection and disposal (Corporation, 2022). In the environmental aspects due to the Covid-19 greenhouse gas (GHG) emissions could drop never before since world-war II (Global Carbon Project, 2020) (Rume and Islam, 2020). The air quality was improved and notable change in the appearance of many beaches in the world. Now it looks cleaner and with crystal clear waters. Noise level dropped considerably in most cities in the world (Zambrano-Monserrate et al., 2020). Collective reports explore the impact of COVID-19 on environment that can be categorize into short term positive impacts (lockdown) and long-term negative impacts. Short term positive impacts are decrease in criteria pollutants, reduced sound pollution, reduced GHG concentration, overall environmental cleanliness, wildlife recurring to lost areas, etc. The long-term negative impacts are high production of plastic waste, recycling rate reduction, increased the level of organic and inorganic pollutants because of soap and hand sanitizers, increased waste water and in future, the water consumption of individual person shall increases (Sharma, 2020).

### 4.3 Domestic waste

The widespread lockdown forces individuals to stay in home which substantially increases the domestic waste generation (Rupani et al., 2020). The generation of large amounts of domestic waste requires collection and recycling, but due to manpower shortages it was compromised in many countries. In UK Interrupted services have led to waste mismanagement increases of 300% in some rural communities. In recent days the urban areas are facing environmental prospects owing to migration and depletion of natural resources as 80–90% of municipal waste is disposed in land filled without proper management with open fire. In term of Municipal Solid Waste Management (MSWM) the challenges in the form of exponential population growth, high density of urban regions, various culture, moving food habits, and recent routines of life style problems are encountered. Meanwhile, MSWM also facing the issues related to the waste collection, treatment and disposal. Notably, the main issues are improper valuation of quantity and quality of solid waste (Kumar et al., 2020). Sudden lockdowns have synchronized with the peak time of harvesting of summer vegetables that cause high impact on economic loss on Indian farmers and that also generate enormous food wastages (Sharma et al., 2020). To fix this issue, Government of India have initiated various measures to supply food for remote areas, operative preservation of food and its fast transmission to the marketplace so as to reduce unparalleled wastage of food (Ganguly, 2021). The crisis triggered by the COVID-19 pandemic in generating the waste has increased immensely and affected SWM in the country and the mixing of virus infected biomedical waste with the stream of normal solid waste has highly major negative safety and health concerns. This pandemic has revealed many lapses and shortcomings across country in managing the solid waste. The air quality has improved but adverse effects associated with the increased production of solid waste and decreased recycling process created long-term impact as improper collection could result in spreading of more virus among the people it was a quite challenging for SWM to safely collect, handle, transport and dispose. As per CPCB guidance double-layered bag is utilized for waste collection from COVID-19 insulation wards to confirm sufficient strength and no leaks and biomedical waste are collected and stored separately at CBWTF before handling. Few techniques to be followed—Incineration, Alternative thermal techniques (Singh, 2022). The coronavirus disease 2019 (COVID-19) pandemic has led to an unexpected collapse of waste management chains (Siming and Ok, 2020). Medical waste like contaminated masks, gloves, medications and etc., can easily be mixed with domestic waste. So safely managing the domestic and medical waste is a crucial step to successfully prevent the disease (Sangkham, 2020). Covid-19 pandemic spread is commonly controlled by applying lockdowns to reduce the spread, whereas, the quantity of waste (household, medical waste, non-medical waste and food waste) is increasing across the world countries (Klemeš et al., 2020; Sarkodie and Owusu, 2021a). As a prevention measure for Covid-19, people preferred to use only single-use products and the usage/percentage of masks, gloves, thermometers, sanitizers and cleaning products, toilet papers and foodstuffs are increasing as waste materials (Sarkodie and Owusu, 2021b). Covid-19 pandemic lockdowns indirectly directing the people to use plastic materials that will influence the plastic usage reduction (Sarkodie and Owusu, 2021a). Importantly, lockdown is the main reason for the supply chain break for an example, during lockdown times the food materials trucks, vehicles carrying vegetables, food material suppliers got stucked and those stock materials had more chances to turn as waste (Sharma et al., 2020). Mismanagement can also lead to increased environmental pollution. Reports says that there is high possibility of COVID-19 spread to the community through the household waste as it stays in the plastic, stainless steel, copper, cardboard and other materials (Kumar et al., 2021a, Kumar et al., 2021b). It is always advisable to keep the waste in a closed garbage bag before its disposable stage. Compared to the other house hold waste materials the medical waste has been increased enormously during this pandemic time. The management of health care waste management using defined steps (moreover similar in worldwide) such as, waste collector should collect the waste as per government guidelines, disinfecting, sorting, temporary storage, composting and incineration and reusing (Das et al., 2021). Notably, the Covid-19 household waste and medical waste should be labelled as Covid-19 waste, following that, the disinfectants (80% ethanol, 75% isopropanol) applied on household medical waste but the medical Covid-19 waste are highly hazardous so that it must be autoclaved. Finally, both wastes will be incinerated and due to this pandemic, the recycling operation unit is less operational for non-Covid-19 waste and Covid-19 house hold waste that are considered as residual waste and medical Covid-19 waste has to be disposed appropriately (A. Tripathi et al., 2020).

Waste Management is one of the most important sanitary wall to prevent the spread of illnesses and diseases. Continuity of the waste services for municipal waste, hazardous industrial and healthcare waste and is probably carried out by the sanitary workers. So, it is must to ensure the health and safety precautions of waste workers by guiding them to frequently change and to clean with professional clothing, replacing professional gloves instantly following breakage or any incident of potential contamination, sanitizing vehicle cabins, regular facilities and other handling equipment’s etc.

## 5 Future perspectives

Integrated Solid Waste Management System hierarchy mostly prefers at source reduction and reuse, recycling, composting, waste to energy conversion and least preference has been given to land filling. The policies should also ensure the following hierarchy—avoid, reuse, recycle, recover and dispose. For waste treatment, characterization of waste and choosing the appropriate techniques is more important example -3Rs strategy and awareness creation will help to reduce the plastic waste Worldwide. India has launched 2016 SWM Rules that address an important number of concerns, compliance remains weak but India has conducted more campaigns like Source Segregation Campaign (part of Swachh Bharat Mission) to motivate people to segregate their waste and it has been successfully achieved in most sections of waste management like waste generation (total wards—84,475 and total waste generation by waste 14, 7613 Mt/d), door to door collection (total average of wards with 100% door to door collection is 81,135 (96.05%) out of 84,7475), segregation (100% waste segregation at household levels in 63,204 wards (74.82%). Ameliorating the creation of inventive alternatives with proper screening methods would improve the step up on bio-waste management. Organic waste shares the more percentage of municipal solid waste which are most probably treated using techniques like Windrow composting, In-vessel composting, Vermicomposting, Black soldier fly processing, anaerobic digestion and slow pyrolysis. Notably, the conversion process of an organic waste into compost and digestate products that can improve the soil growth and quality using closed biological cycles. Emerging technologies and advancements in each phase of solid waste management have been reported but the availability of technological updates in the waste management system is not sufficient. SWM system in India is in a critical stage because it mainly fails to manage solid waste management in urban local bodies. In future, emerging new technologies are essential to reach a level that can manages waste in a cheaper way than dumping it. As a future solution what need to be done is critical to think and in present condition most of the developed countries evolved management plans for the next 30–50 years. The key motto is to “Bring the waste under control” for that four-action plan are formulated that will keep the waste management to a high standard in future. 1. Stop uncontrolled waste dumping and burning—which ensure collection coverage and controlled disposal. 2. Holistic approach for hazardous waste—Treatment plant at the source site and managing them separately to facilitate controlled environment. 3. Focus primarily on waste prevention—maximize usage by reuse and recycling. 4. Focus on the feedback loop—integrating recycling with the mainstream of waste management and developing sound techniques for energy recovery. Citizen participation has to be promoted and prominently in source segregation and treatment processes in India to make an efficient solid waste management system. Significantly, the policy agenda has to be made to grow up the attitude of citizens, decision-makers, reduce the level of wastages and littering and ameliorate the process of reuse and recycle. But notably, plastic is being a tough fighter for solid waste management, ameliorating the alternatives (edible bio plastics, bagasse, palm leaves, mushroom root) easily degradable polymeric materials should be encouraged by government and people effectively.

## 6 Conclusion and prospects

Solid waste is continuously increasing due to increasing population, urbanization and would be a biggest challenge due to its direct impact on public health and environment. In present scenario, waste collection, treatment and disposal procedures are to be upgraded for efficient management of solid waste. Lack of waste disposal facilities and unmanaged collection coverage are the major problems faced in developing countries which causes impact on environmental contamination. To ameliorate the numbers of trainees and proper awareness for the workers are an inevitable step to achieve the successful waste collection process. In training the front-line workers should be actively involved in assigned work with commitments which can be inherited through proper caring the workers by adopting labour welfares, hazard pay, sick leave, insurance, providing additional equipment’s. Vehicle drivers should be trained in such a way to avoid accidents, obeying speed limit and gentle handling of brakes will increase fuel efficiency economically by 15–30% in highways (US department of Energy—Conserve Fuel), safe as well as speed up in the collection process. Proper communication and understanding between the driver and waste collector is essential to reduce delays as well as recent contexts like choosing the motorized or non-motorized vehicle will help to monitor waste collection. Choosing the right motorized or non-motorized vehicle based on street width aid to cover the inaccessible area. GPS enabled vehicle, Weigh Bridge, electronic on-board recorders, street camera, staff training, communication through technology are some of the recent advancements that can help to monitor waste collection process. The demand for landfilling space for handling solid waste disposal is also an essential factor in solid waste management system and that has to be focused worldwide. More importantly, collective reports explore some of the points which are very essential for solid waste management systems such as, the maximum quantity of waste (450–500 million tons) generated is reported from East Asia and Pacific regions. Meanwhile, except Central Asia, other Asian countries recycling rates are below 10% that should be taken as a serious concern to prevent the effects. Worldwide 33% of waste is disposed by open dump method and out of that only 13.5% of waste treated for recycling process. But positively some countries have high concern in recycling and 3Rs technology such as Australia have investing 42% of money on recycling and Japan has investing 80% of their money in waste incineration process that are welcoming and inspiring other countries.

## Declarations

### Author contribution statement

All authors listed have significantly contributed to the development and the writing of this article.

### Funding statement

This research did not receive any specific grant from funding agencies in the public, commercial, or not-for-profit sectors.

### Data availability statement

No data was used for the research described in the article.

### Declaration of interest’s statement

The authors declare no conflict of interest.

### Additional information

No additional information is available for this paper.
